# Lymphatic exosomes promote dendritic cell migration along guidance cues

**DOI:** 10.1083/jcb.201612051

**Published:** 2018-06-04

**Authors:** Markus Brown, Louise A. Johnson, Dario A. Leone, Peter Majek, Kari Vaahtomeri, Daniel Senfter, Nora Bukosza, Helga Schachner, Gabriele Asfour, Brigitte Langer, Robert Hauschild, Katja Parapatics, Young-Kwon Hong, Keiryn L. Bennett, Renate Kain, Michael Detmar, Michael Sixt, David G. Jackson, Dontscho Kerjaschki

**Affiliations:** 1 Clinical Department of Pathology, Medical University of Vienna, Vienna, Austria; 2 Institute of Science and Technology, Klosterneuburg, Austria; 3 Medical Research Council Human Immunology Unit, Weatherall Institute of Molecular Medicine, John Radcliffe Hospital, University of Oxford, Oxford, England, UK; 4 CeMM Research Center for Molecular Medicine of the Austrian Academy of Sciences, Vienna, Austria; 5 Department of Pediatrics and Adolescent Medicine, Medical University of Vienna, Vienna, Austria; 6 Norris Comprehensive Cancer Center, University of Southern California, Los Angeles, CA; 7 Institute of Pharmaceutical Sciences, Swiss Federal Institute of Technology, ETH Zurich, Zurich, Switzerland

## Abstract

Lymphatic endothelial cells (LECs) release extracellular chemokines to guide the migration of dendritic cells. In this study, we report that LECs also release basolateral exosome-rich endothelial vesicles (EEVs) that are secreted in greater numbers in the presence of inflammatory cytokines and accumulate in the perivascular stroma of small lymphatic vessels in human chronic inflammatory diseases. Proteomic analyses of EEV fractions identified >1,700 cargo proteins and revealed a dominant motility-promoting protein signature. In vitro and ex vivo EEV fractions augmented cellular protrusion formation in a CX3CL1/fractalkine-dependent fashion and enhanced the directional migratory response of human dendritic cells along guidance cues. We conclude that perilymphatic LEC exosomes enhance exploratory behavior and thus promote directional migration of CX3CR1-expressing cells in complex tissue environments.

## Abstract (precis)

Inflammation stimulates lymphatic endothelial cells to release exosomes, which accumulate in the perivascular stroma. Brown et al. show that these exosomes promote the directional migration of dendritic cells along guidance cues in complex environments by enhancing dynamic cellular protrusions in a CX3CL1-dependent manner.

## Introduction

The interstitial space contains membrane vesicles (Yáñez-Mó et al., 2015; Weigelin et al., 2016), which according to their biogenesis, are commonly classified as apoptotic bodies, ectosomes, and exosomes. Exosomes are characterized by a diameter of 30–100 nm (maximum 150 nm as seen by EM; Colombo et al., 2014), a buoyancy of 1.16–1.23 g/ml in density gradient centrifugation (Théry et al., 2006; Lobb et al., 2015), and a protein fingerprint that is compiled in the ExoCarta consensus database (Keerthikumar et al., 2016) and is constantly expanded and revised (Kowal et al., 2016). The cargo of exosomes consists of proteins, mRNAs, and microRNAs, and their membranes are rich in cholesterol, phosphatidylserine, and ceramide (Yáñez-Mó et al., 2015). These vectorial signalosomes are produced in multivesicular bodies (MVBs) of a wide range of cells and serve as potential long- and short-range communicators (Harding et al., 1984; Johnstone et al., 1984; Yáñez-Mó et al., 2015). Exosomes transfer numerous factors that promote cell migration in auto- and paracrine modes (Truman et al., 2008; Baj-Krzyworzeka et al., 2011; Sung et al., 2015; Majumdar et al., 2016) and are potentially relevant for immune and cancer cell migration (Hoshino et al., 2015; Wendler et al., 2017). En route to their target organs migratory cells have to overcome various architectural hindrances, including interstitial matrix structures, basement membranes, and intercellular junctions. To navigate through such complex environments, migrating cells have to maintain dynamic cellular protrusions, which constantly sample and explore the chemical and geometrical features of their surroundings for guidance (Leithner et al., 2016).

During inflammatory conditions, several cell types, including dendritic and cancer cells, cross endothelial barriers to enter the lumen of small blood and lymphatic vessels. Therefore, exosomes released by endothelial cells would be in a favorable position to affect the migratory pathways of incoming cells. Endothelial cells of the blood vasculature were shown to produce extracellular vesicles during steady-state (ss; van Balkom et al., 2013), hypoxic (Umezu et al., 2014; Mayo and Bearden, 2015; de Jong et al., 2016), apoptotic (Dieudé et al., 2015), inflammatory (Walker et al., 2009; Yamamoto et al., 2015), and angiogenic (Sheldon et al., 2010) conditions. To date however, the possibility of exosome secretion by lymphatic endothelial cells (LECs) has not yet been explored, although recent findings point to a major role of lymphatic vessels in transporting exosomes (Srinivasan et al., 2016). Moreover, a role for exosomes within the lymphatics might well be envisaged in clearing cells from sites of resolving inflammation (Ranghino et al., 2015) and guiding dendritic and other immune cells along chemokine gradients into lymph nodes to mount an appropriate immune response (Heuzé et al., 2013; Weber et al., 2013; Russo et al., 2016; Johnson et al., 2017). Previous work has highlighted the in vivo importance of the CX3CL1–CX3CR1 signaling axis for lymphatic trafficking of dendritic cells under conditions of inflammation (Johnson and Jackson, 2013) and its exploitation by cancer cells for metastasis (Shulby et al., 2004; Andre et al., 2006; Marchesi et al., 2008; Castellana et al., 2009; Locatelli et al., 2010; Yao et al., 2014; Shen et al., 2016). In this study, we found that the exosome marker proteins tetraspanin 29 (CD9) and tetraspanin 30 (CD63) accumulate around lymphatic vessels, particularly in inflamed and cancerous tissues. Furthermore, we demonstrate that exosome-rich endothelial vesicle (EEV) fractions are increasingly released in vitro by human LECs after exposure to an inflammatory cytokine (TNFα). State-of-the-art quantitative proteomic analysis of EEV fractions from primary human LECs revealed the presence of >1,700 cargo proteins. Alongside several endothelial marker proteins, the dominant abundance of chemokines and growth factors, actin cytoskeleton (regulatory) proteins, motor proteins, adhesion proteins, and proteolytic enzymes in inflammatory EEV fractions were indicative of a motility-promoting function. We found that exposure to inflammatory EEV fractions induced dynamic cellular protrusion formation of mature human monocyte-derived dendritic cells (MMDCs) via surface-bound CX3CL1/fractalkine. Accordingly, in vitro and ex vivo inflammatory EEV fractions increased the directional migratory response of motile CX3CR1^+^ cells along soluble chemical and geometrical guidance cues in complex environments.

## Results

### Immunohistological evidence for exosome accumulation in the perilymphatic stroma of inflamed tissues

**Figure 1.**
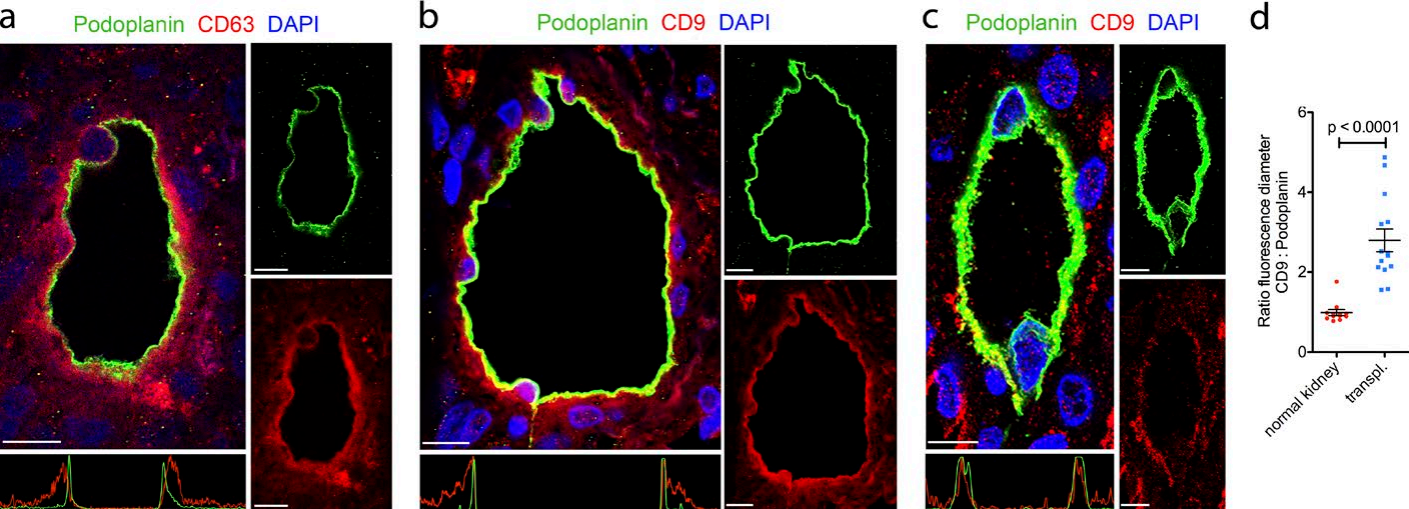
**Immunohistological evidence for exosome accumulation in the perilymphatic stroma of inflamed human kidneys. (a and b)** Immunofluorescence of the lymphatic vessel marker podoplanin (green) and the exosome markers CD9 (a, red) or CD63 (b, red) in human renal transplant rejections (fluorescence profiles across respective vessels are plotted beneath merged images; x axis: cross-sectional distance; y axis: CD9-, CD63-, or podoplanin-specific mean immunofluorescence intensities). Cell nuclei are stained with DAPI (blue; *n* = 14). **(c)** Immunofluorescence of the lymphatic vessel marker podoplanin (green) and the exosome marker CD9 (red) in normal human kidneys (fluorescence profile across respective vessel is plotted beneath merged images; x axis: cross-sectional distance; y axis: C9- or podoplanin-specific mean immunofluorescence intensities). Cell nuclei are stained with DAPI (blue; *n* = 12). Bars, 10 µm. **(d)** Ratios of fluorescence diameters of CD9/podoplanin stainings of lymphatic vessels in normal (*n* = 12) and rejecting transplanted human kidneys (*n* = 14). Data obtained from at least two lymphatic vessels per patient sample and from six different patients who were pooled for analysis (unpaired two-tailed *t* test with Welch’s correction). Values represent means ± SEM.

To evaluate the potential presence of exosomes in human and mouse tissues we immunostained a range of normal and chronically inflamed tissues with antibodies against the exosome markers CD9 and CD63, the lymphatic endothelial marker podoplanin, and the panvascular marker platelet endothelial cell adhesion molecule (PECAM1/CD31). In tissue sections from patients with inflammatory conditions, including renal transplant rejection (Fig. 1, a and b), allergic dermatitis (Fig. S1 a), Crohn’s disease (Fig. S1 b), and oxazolone-induced hypersensitivity dermatitis of mice (Fig. S1 c), lymphatic vessels were surrounded by basolateral “halos” of exosome markers. The immunoreactivity for CD9 and CD63 was increased in LECs of inflamed kidneys, and the diameters of the perivascular halos were enlarged 2.8-fold (P < 0.0001) compared with control tissues (Fig. 1, c and d; and Fig. S1 d). Notably, the perivascular stroma of CD31^+^ blood vessels in chronically inflamed renal transplants was devoid of exosome marker accumulation (Fig. S1 e).

### LECs release EEVs

**Figure 2.**
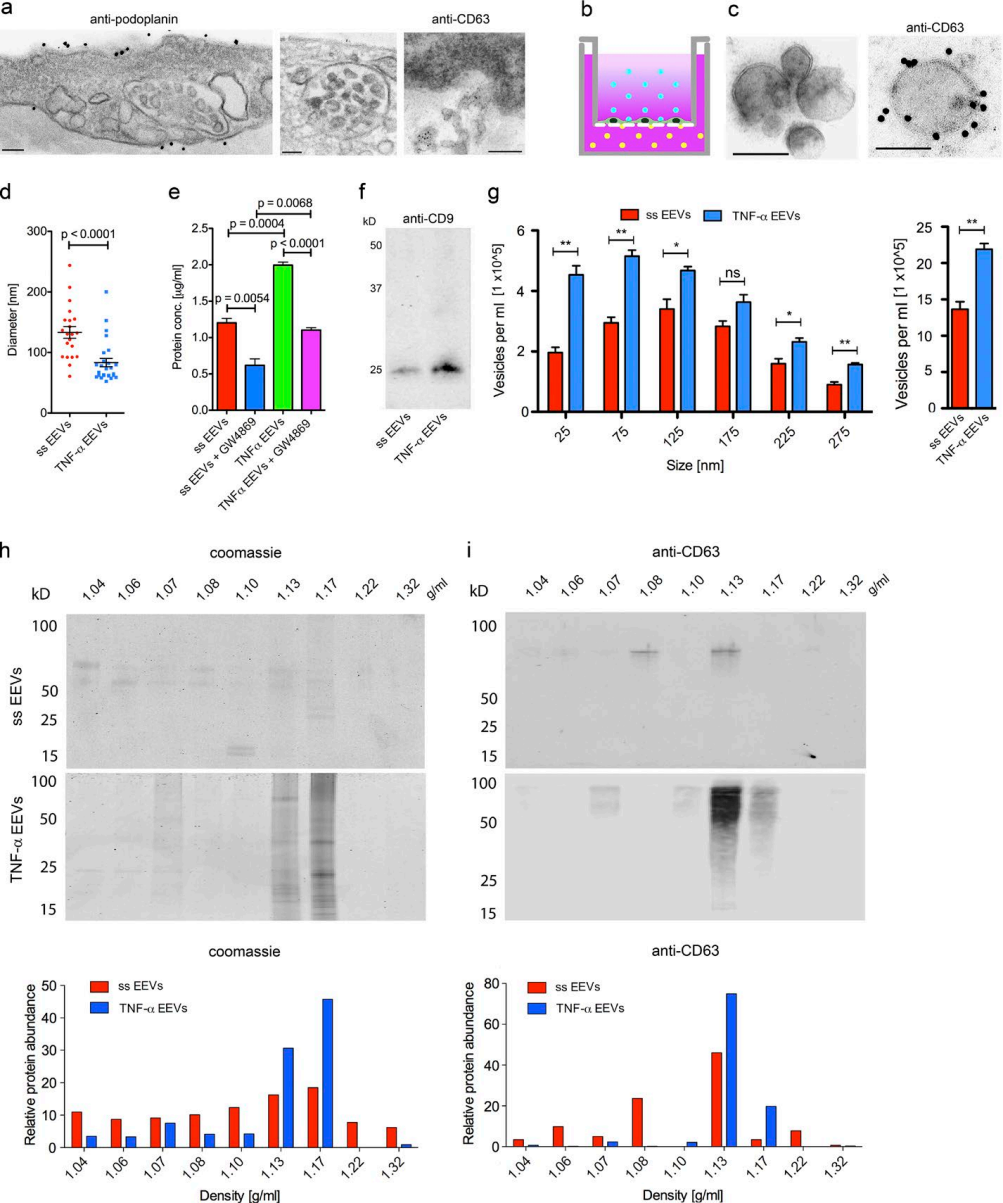
**Primary human LECs release EEVs. (a)** Transmitted EM (TEM) of human skin LECs in situ (left: antipodoplanin-conjugated gold particles) and in vitro (middle and right). MVBs (middle, unstained) and exocytosis of CD63^+^ vesicles (right, anti-CD63–conjugated gold particles) into the basolateral stroma (*n* ≥ 5). **(b)** Experimental setup to collect basolateral LEC culture supernatant from the lower chamber for the enrichment of basolateral EEVs (yellow dots). **(c)** TEM of vesicles of basolateral EEV fractions. Right: Anti-CD63–conjugated gold particles (*n* ≥ 21). Bars, 100 nm. **(d)** Diameters of vesicles in basolateral EEV fractions collected from ss (*n* = 21) and TNFα-stimulated LECs (*n* = 26). Data are obtained from at least seven TEM images per experiment and from three independent experiments that were pooled for analysis (unpaired two-tailed *t* test with Welch’s correction). **(e)** BCA-derived protein concentrations of EEV fractions collected over 24 h from ss and TNFα-stimulated LECs in the absence or presence of 10 µM GW4869 (*n* = 3; unpaired two-tailed *t* test). **(f)** Anti-CD9 immunoblot of EEV fractions from equal volumes of basolateral ss and TNFα-stimulated LEC culture supernatants (*n* = 3). **(g)** Mean absolute vesicle numbers of EEV fractions isolated from 1 ml basolateral LEC culture supernatant of ss and TNFα-stimulated LECs (*n* = 3; unpaired two-tailed *t* test). Left graph, vesicle numbers plotted against diameter size intervals; right graph, vesicle numbers of all sizes. **(h)** Coomassie blue–stained electrophoresis gel (top) and quantitation of relative protein abundance (as measured by densitometric analyses; bottom) of density gradient centrifugation subfractions of EEV fractions isolated from basolateral culture supernatants of ss and TNFα-stimulated LECs (*n* = 3). **(i)** Anti-CD63–stained immunoblot (top) and quantitation of relative protein abundance (as measured by densitometric analyses; bottom) of density gradient centrifugation subfractions of EEV fractions isolated from basolateral culture supernatants of ss and TNFα-stimulated LECs (*n* = 3). Values represent means ± SEM. ns, P > 0.05; *, P ≤ 0.05; **, P ≤ 0.01.

To determine whether the lymphatic endothelium could be a source of the putative exosome halos, we imaged the ultrastructure of human dermal LECs in vitro and in situ (Fig. 2 a). MVBs, the source of exosomes, were present in LECs, and CD63^+^ vesicles were captured in the process of their basolateral release into the extracellular space. This prompted us to analyze the vesicular content of basolateral supernatants from confluent primary human dermal microvascular LEC monolayers (Figs. 2 b and S2 a). Vesicular fractions were enriched by the Exoquick-TC exosome precipitation protocol and hence designated as EEVs. The isolated vesicles were immunoreactive for CD63 (Fig. 2 c), and their diameters averaged 133 ± 9.6 nm under ss conditions (Fig. 2 d). To mimic inflammatory settings in vitro we incubated cultured LECs with TNFα before harvesting vesicle fractions as described previously (Johnson and Jackson, 2013). TNFα increased the protein concentration of EEV fractions (TNFα-EEVs) from basolateral LEC culture supernatants by 1.66-fold (P = 0.0004) over ss-EEV fractions (Fig. 2 e). The diameters of TNFα-EEVs were smaller (83 ± 6.9 nm) and more closely matched the reported diameters (30—100 nm) for exosomes than those of ss-EEVs (Fig. 2 d; Colombo et al., 2014). Densitometry of immunoblots showed a 1.58-fold increase of CD9 in TNFα-EEV fractions compared with ss-EEV fractions (Figs. 2 f and S2 b). The TNFα-specific increase in protein concentration was accompanied by a 1.60-fold (P = 0.0032) increase in vesicle numbers in TNFα-EEV fractions as measured by NanoSight tracking analysis (NTA; Fig. 2 g). Subfractionation of EEVs by density gradient centrifugation and subsequent analyses by SDS-PAGE, Coomassie blue staining, and immunoblotting for CD63 revealed that 35% of ss-EEVs and as much as 76% of TNFα-EEVs floated at an exosome (CD63^+^)-specific density of 1.13 to 1.17 g/ml (Fig. 2, h and i; and Fig. S2, c and d). Indeed, pretreatment with GW-4869, an inhibitor of neutral sphingomyelinase-2, the enzyme that controls the intraluminal budding of vesicles into MVBs and determines the number of exosomes (Trajkovic et al., 2008), reduced the protein content of ss- and TNFα-EEV fractions by 50% and 45%, respectively (Fig. 2 d).

### Proteomic profiling reveals a migration-promoting protein signature in TNFα-EEV fractions

**Figure 3.**
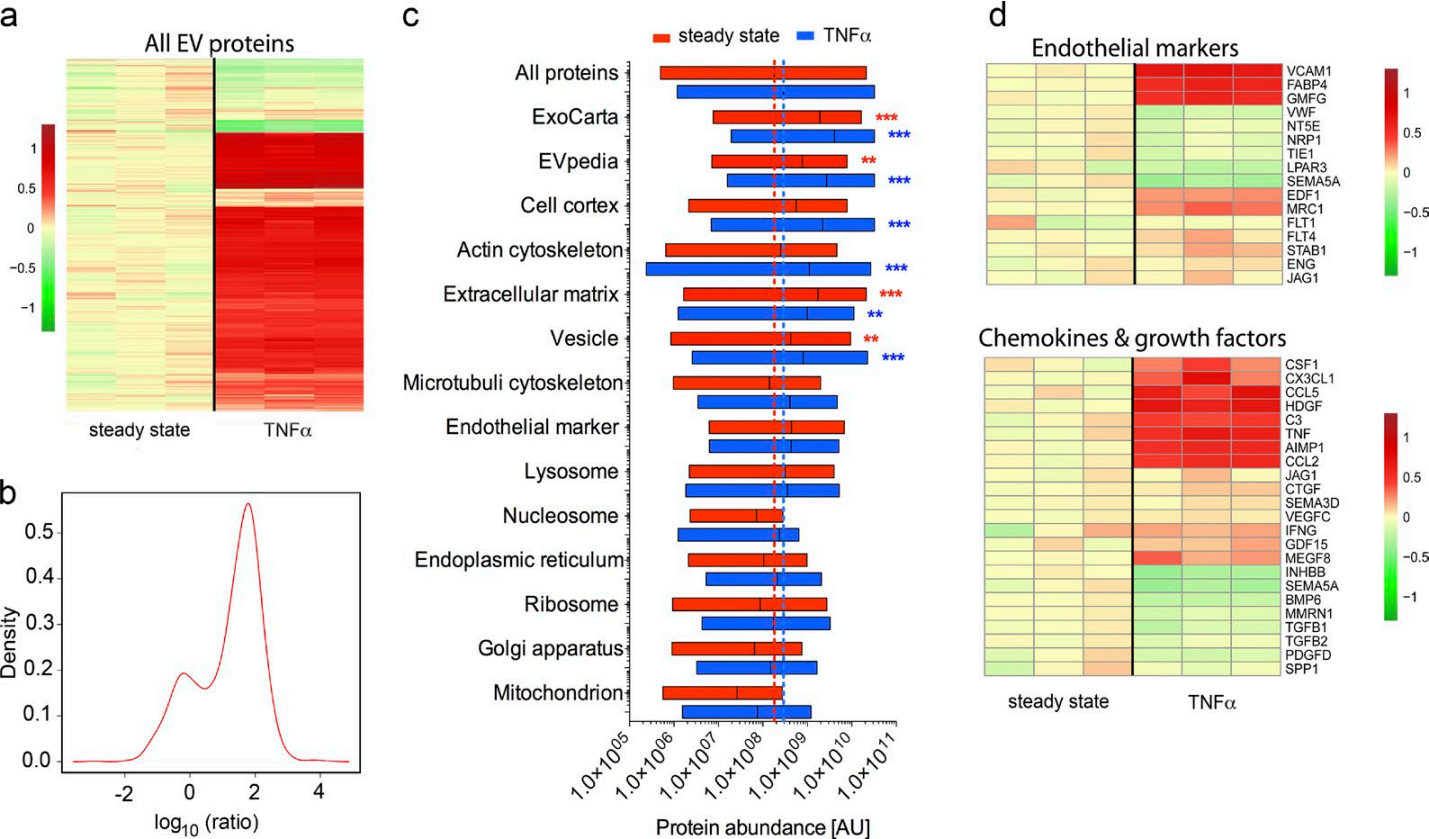
**Proteomic profiling reveals a migration-promoting protein signature in TNFα EEV fractions. (a–d)** EEV fractions from basolateral culture supernatants of ss (*n* = 3) or TNFα-stimulated LECs (*n* = 3) were proteomically profiled with TMT-based LC-MSMS analysis. **(a)** Heat map of all significantly (P < 0.05) quantified proteins of EEV fractions. Three left lanes: Replicate EEV fractions from ss LECs; three right lanes: Replicate EEV fractions from TNFα-stimulated LECs. Values in heat maps are log_10_ of interprotein abundance of a given sample divided by the mean abundance of the three ss samples. **(b)** Ratio density plot of EEV fractions from ss and TNFα-stimulated LECs. X axis: Log_10_ ratio of interprotein abundance of TNFα-EEV fractions over ss-EEV fractions. Y axis: Relative number of proteins. **(c)** The quantitatively identified proteins of EEV fractions were grouped into biologically relevant clusters according to the databases of ExoCarta (exosomes), EVpedia (extracellular vesicles), and the Gene Ontology Consortium (cellular components). Mean interprotein abundances of proteins in the respective clusters were compared with the mean interprotein abundance of all proteins in ss-EEV fractions (red dotted line) and TNFα-EEV fractions (blue dotted line). X axis: Logarithmic scale of interprotein abundances. Y axis: Biologically relevant clusters. For each cluster, the floating bars display the minimum-to-maximum intervals of the interprotein abundance for ss-EEV (red) and TNFα-EEV (blue) fractions. Vertical lines show the mean interprotein abundance for ss-EEV (red) and TNFα-EEV (blue) fractions. **, P < 0.01; *** P ≤ 0.001. **(d)** Heat maps of significantly (P < 0.05) quantified proteins of EEV fractions that are either endothelial markers or chemokines and growth factors.

To confirm the exosomal identity of EEV fractions and to resolve their potential biological functions, we performed proteomic profiling. Analyses of ss-EEV fractions and TNFα-EEV fractions from basolateral LEC culture supernatants by tandem mass tag (TMT)-based quantitative liquid chromatography–tandem mass spectrometry (MS; LC-MSMS) resulted in the identification of 1,717 proteins, of which 1,384 were significantly (P < 0.05) up- or down-regulated by TNFα stimulation (Table S1). The set of regulated proteins included 117 transmembrane proteins, 152 secreted proteins, 51 ECM proteins, 724 cytoplasmic proteins, and 84 transcription factors. Compared with ss-EEV fractions, TNFα-stimulation increased the abundance of 1,203 proteins in EEV fractions by at least 1.5-fold, whereas 84 were down-regulated by at least 1.5-fold (Fig. 3 a). Consistent with these results, ratio–density analysis revealed a 1.7-fold up-regulation of the overall protein abundance in EEV fractions upon TNFα-treatment (Fig. 3 b). Importantly, the magnitudes of the majority of protein up-regulations correlated with the enhanced secretion levels of vesicle numbers, indicating that the inflammatory cytokine changed primarily the amount of extracellularly released vesicles, rather than their protein content (Fig. 2 g).

The proteomic profile of EEV fractions comprised 24 of the top 25 marker proteins listed in the extracellular vesicle compendium EVpedia (Kim et al., 2015), 23 of 25 exosome-specific proteins in the ExoCarta database (Fig. S3; Keerthikumar et al., 2016), and 746 of 5,071 extracellular exosome proteins from the Gene Ontology Consortium database (Fig. 3 c; Ashburner et al., 2000). In particular, the exosome-biogenesis proteins (Fig. S3) programmed cell death 6-interacting protein (ALIX; Geeraerts et al., 2013; Friand et al., 2015) and CD63 (Andreu and Yáñez-Mó, 2014) were increased 2.93- and 1.45-fold, respectively, in TNFα-EEV fractions.

Grouping identified proteins into biologically relevant clusters and comparing the mean interprotein abundances of clusters with the mean interprotein abundance of all identified proteins revealed that the ExoCarta protein cluster was 10.55-fold enriched (Fig. 3 c) in ss-EEV fractions and as much as 14.03-fold enriched in TNFα-EEV fractions. Similarly, the EVpedia cluster was 4.22-fold enriched in ss-EEV fractions and 9.36-fold in TNFα-EEV fractions (Fig. 3 c). Notably, EEV fractions lacked any significant contamination by other cellular components such as intracellular organelles (Fig. 3 c). Together with the data on vesicle size, their buoyancies in density gradient centrifugation and their neutral sphingomyelinase-dependent biogenesis, these results indicate a high exosome purity in EEV fractions, which was especially pronounced in TNFα-EEV fractions.

In accordance with their cellular origin, EEV fractions were positive for established endothelial marker proteins (Fig. 3 d) and, intriguingly, carried numerous motility promoting proteins such as actin cytoskeleton (regulatory) proteins (Figs. 3 c and S3), chemokines and growth factors (Fig. 3 d), cytoskeletal motor proteins (Fig. S3), cell adhesion molecules (Fig. S3), and several proteolytic enzymes (Fig. S3). Because inflammatory settings appeared to cause the basolateral endothelial secretion and accumulation of exosomes in the perivascular stroma of lymphatic vessels, the latter results raised the question of whether LEC exosomes could modulate the migration of dendritic cells that traffic via the lymphovascular system.

### In vitro and ex vivo EEV fractions promote the directional migration of MMDCs along guidance cues

**Figure 4.**
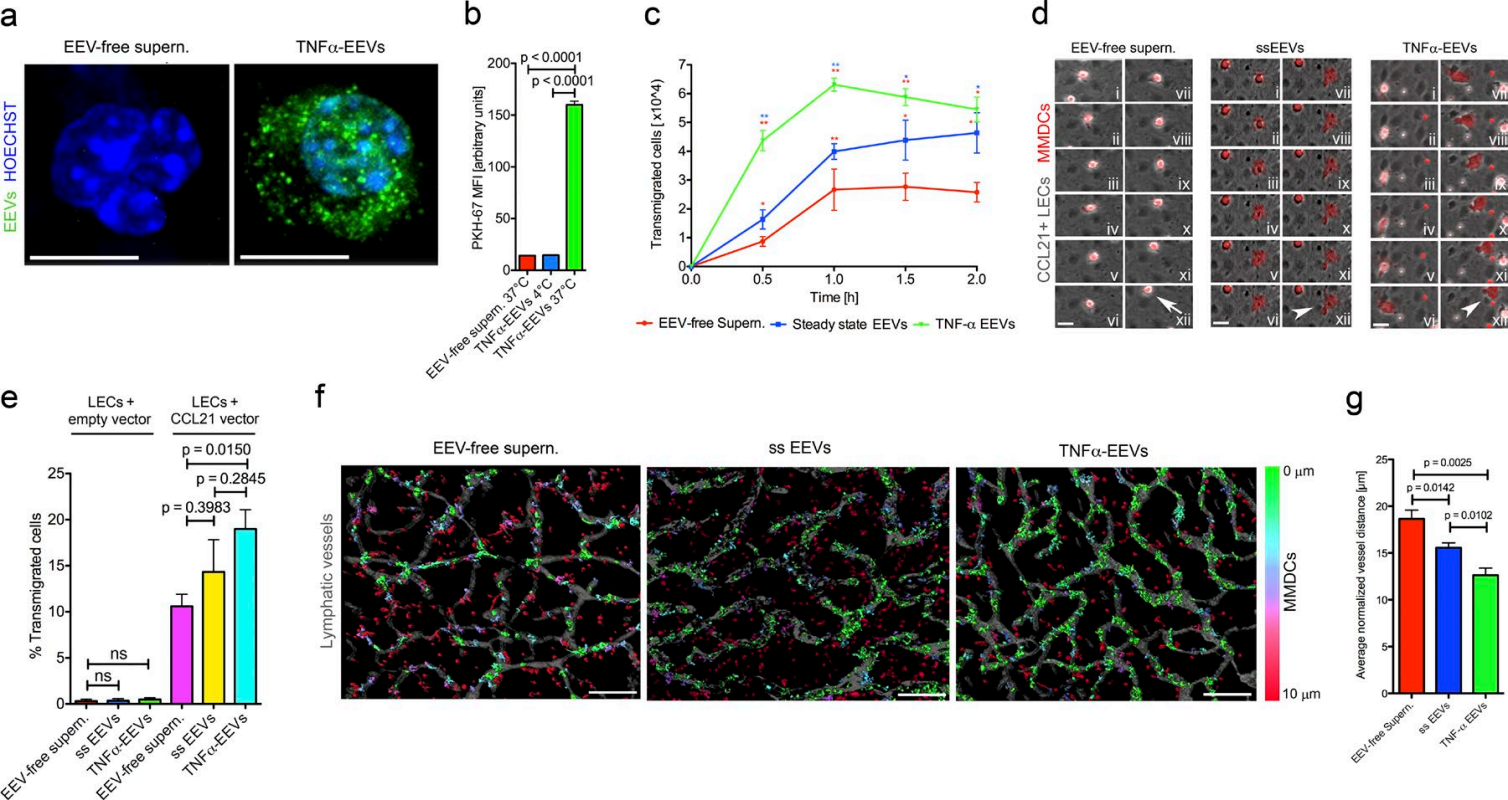
**EEV fractions promote the directional migration of human matured MMDCs along guidance cues in vitro and ex vivo. (a)** Fluorescence images of Hoechst-stained (blue) MMDCs that were embedded in a 3D collagen matrix that contained SYTO RNASelect–stained TNFα-EEV fractions (green, left) or SYTO RNASelect–stained EEV-free supernatants (green, right; *n* = 5). Bars, 5 µm. **(b)** Flow cytometry analysis of MMDCs incubated for 60 min with PKH67-stained EEV-free supernatants or PKH67-stained TNFα-EEV fractions at 37°C or 4°C (*n* = 3; unpaired two-tailed *t* test). **(c)** Quantitation of MMDCs that directionally transmigrated through the porous membrane of the upper cell culture insert into the lower chamber well of a transwell assay. MMDCs were loaded into the upper cell culture insert after the lower well chamber was loaded with EEV-free supernatants, ss-EEV fractions, or TNFα-EEV fractions (*n* = 3; unpaired two-tailed *t* test). **(d)** Time-lapse image series (first frame top left image [i], last frame bottom right image [xii]) over 40 min of fluorescent MMDCs transmigrating a CCL21-overexpressing LEC monolayer in the presence of EEV-free supernatants (left), ss-EEV fractions (middle), or TNFα-EEV fractions (right). Arrows mark nontransmigrated MMDCs, and arrowheads mark transmigrated MMDCs (*n* ≥ 4). Bars, 15 µm. **(e)** Relative quantitation of MMDCs transmigrating a CCL21-overexpressing or -nonoverexpressing LEC monolayer in the presence of EEV-free supernatants, ss-EEV fractions, or TNFα-EEV fractions (*n* ≥ 4; unpaired two-tailed *t* test with Welch’s correction). **(f)** Fluorescence images of a split-ear crawl-in assay. Fluorescently labeled MMDCs (color coded) were allowed to migrate for 3 h toward exposed dermal fluorescent lymphatic vessels (gray) in the presence of EEV-free supernatants, ss-EEV fractions, or TNFα-EEV fractions. Color coding indicates the minimum distance of MMDCs to the nearest lymphatic vessel (*n* = 4). Bars, 200 µm. **(g)** Mean minimum distances between the nearest lymphatic vessels and MMDCs (described in f) in the presence of EEV-free supernatants, ss-EEV fractions, or TNFα-EEV fractions were calculated and normalized to the overall vessel density (*n* = 4; unpaired two-tailed *t* test). Values represent means ± SEM. *, P ≤ 0.05; **, P ≤ 0.01.

To explore the interaction between EEVs and dendritic cells in vitro we labeled TNFα-EEV fractions with the membrane-permeable RNA-specific fluorescence dye SYTO RNASelect and incubated them with mature MMDCs in a 3D collagen matrix (Fig. 4 a). EEVs were readily taken up by MMDCs and appeared mainly as vesicular cytoplasmic structures. This uptake was further confirmed by labeling the membranes of TNFα-EEVs with the lipophilic fluorescent dye PKH67 and incubating them with MMDCs. Subsequent FACS analysis revealed a robust uptake of EEVs by the MMDCs at 37°C but no uptake or binding at 4°C (Figs. 4 b and S4, a and b).

Because the EEV cargo included a plethora of motility enhancing proteins, we investigated the ability of EEV fractions to promote migration of MMDCs in transwell migration assays (Fig. 4 c). MMDCs (mean size, 17 µm) were loaded into the upper transwell cell culture inserts and challenged to migrate through 10-µm-thick polyethylene membranes with 3 ± 0.75-µm wide pores separated by an mean distance of 52.63 µm (covering 5.7% of the membrane's area). EEV fractions or EEV-free supernatants were loaded into the lower chambers (Johnson and Jackson, 2013). The migration-promoting effect of EEV fractions was highest within the initial 30 min when the number of migrated cells was increased 1.87-fold (P = 0.0291) by ss-EEV fractions compared with EEV-free supernatant. TNFα-EEV fractions increased the rate of migration 5.00-fold (Fig. 4 c; P = 0.0017). Interestingly, a similar increase of transmigrated MMDCs was obtained when TNFα-EEV fractions were mixed with cells in the upper transwell cell culture insert (Fig. S4 c), indicating that EEV fractions act on MMDCs in a chemokinetic rather than a chemotactic fashion.

We next tested the capacity of EEV fractions to promote the in vitro transmigration of MMDCs through confluent monolayers of primary human LECs (Vaahtomeri et al., 2017). In vivo*,* it is well documented that chemoattraction and transmigration of dendritic cells are dependent on the directional chemical guidance by CC-chemokine ligand 21 (CCL21; Weber et al., 2013), which is secreted by LECs, primarily from their basolateral surface. Because primary human LECs quickly lose CCL21 expression during in vitro culture (Wick et al., 2007; Johnson and Jackson, 2010), MMDCs were unable to transmigrate LECs in the sole presence of EEV-free supernatants, ss-EEV fractions, or TNFα-EEV fractions (Fig. 4, d and e). However, upon reexpression of CCL21 in LECs, the presence of EEV-free supernatants elevated the transmigration of MMDCs by 35.3-fold (P = 0.0002; Video 1), and importantly, the latter was further increased by 1.35-fold in the presence of ss-EEV fractions (P = 0.3983; Video 2) and 1.79-fold in the presence of TNFα-EEV fractions (P = 0.015; Fig. 4, d and e; Video 3). These results indicated that EEVs cooperate with CCL21 to promote directional MMDC transmigration through endothelial monolayers.

To investigate the promigratory EEV effect in a complex native tissue environment, we challenged MMDCs to directionally migrate toward exposed lymphatic vessels in an ex vivo split-ear crawl-in assay in the presence or absence of EEV fractions. In comparison with EEV-free supernatants, ss-EEV fractions and TNFα-EEV fractions increased the movement of MMDCs toward lymphatic vessels by 1.20-fold (P = 0.0142) and 1.48-fold (P = 0.0025), respectively (Fig. 4, f and g). Collectively, these results suggested that exosomes released by inflammatory LECs and forming extracellular perivascular halos promote the migration of dendritic cells in tissues.

### EEV fractions enhance MMDC directional migration in response to guidance cues by inducing formation of cellular protrusions

**Figure 5.**
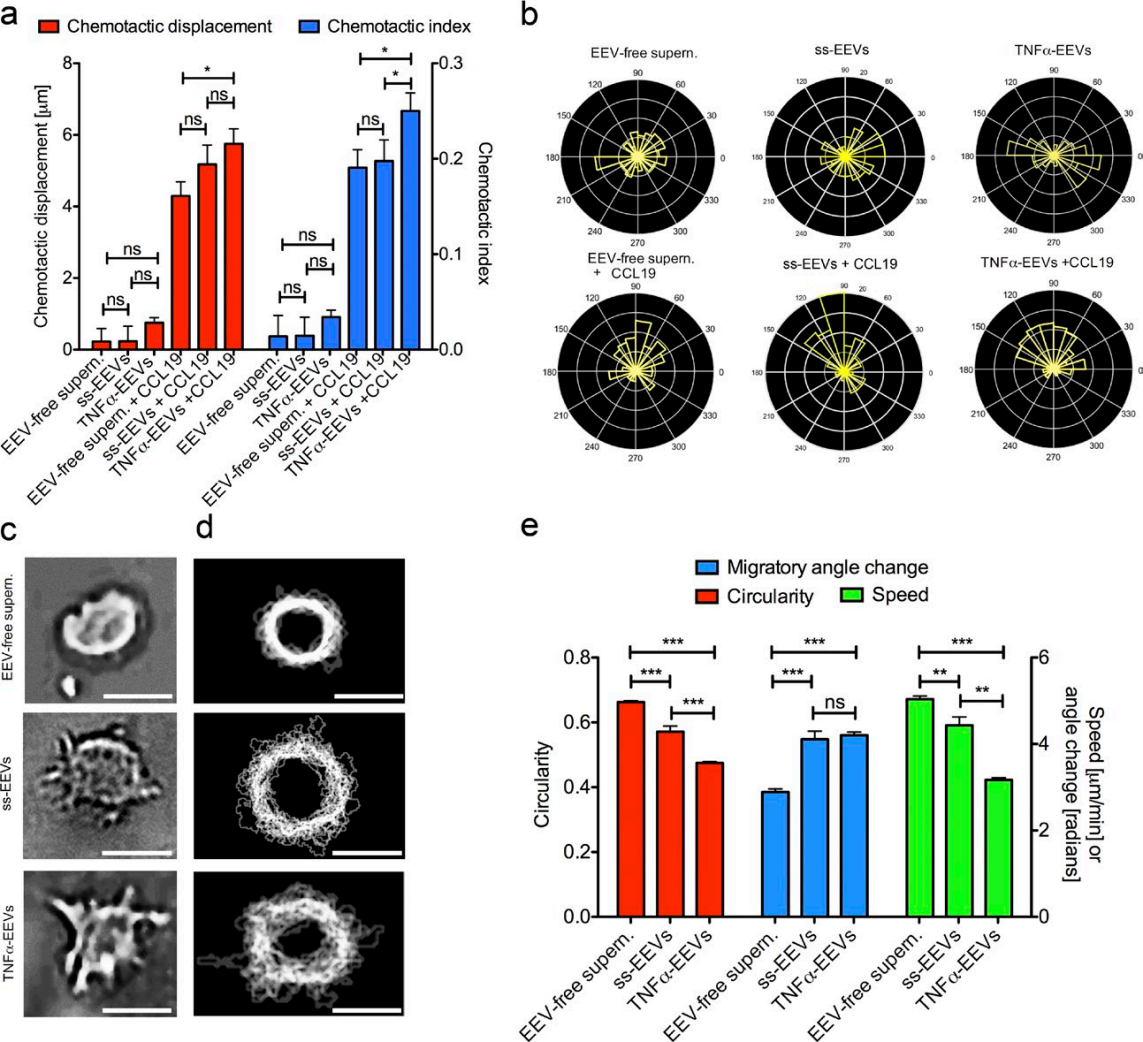
**EEV fractions enhance human matured MMDC directional migration in response to guidance cues by inducing formation of cellular protrusions. (a)** Migration analyses of MMDCs in a 3D collagen matrix migration assay. Cells were exposed to gradients of EEV-free supernatants (*n* = 1,706), ss-EEV fractions (*n* = 2,236), TNFα-EEV fractions (*n* = 8,195), EEV-free supernatants plus CCL19 (*n* = 1,549), ss-EEV fractions plus CCL19 (*n* = 1,833), or TNFα-EEV fractions plus CCL19 (*n* = 1,266). Red columns indicate chemotactic displacement. Blue columns indicate chemotactic index. Data are obtained from at least 349 cell tracks per experiment and three independent experiments that were pooled for analysis (unpaired two-tailed *t* test with Welch’s correction). **(b)** Rose plots representing the angles of migratory tracks of MMDCs described in Fig. 4 a. The yellow columns indicate the relative number of cell tracks that follow a given migratory angle interval. **(c)** Brightfield images of MMDCs migrating in a confined microenvironment migration assay (6-µm height) in the presence of EEV-free supernatants (top), ss-EEV fractions (middle), or TNFα-EEV fractions (bottom; *n* ≥ 21). **(d)** In silico segmented and automatically overlaid cell contours of time-lapse image series of MMDCs described in Fig. 4 c. Images were acquired every 1 min for up to 360 min. Bars: (c) 10 µm; (d) 5 µm. **(e)** Migration analyses of MMDCs in a confined microenvironment migration assay in the presence of EEV-free supernatants (*n* = 921), ss-EEV fractions (*n* = 482), or TNFα-EEV fractions (*n* = 1,632). Red columns indicate circularity of cell shape. Blue columns indicate migratory angle change. Green columns indicate speed of migration. Data are obtained from at least 186 time points from three independent experiments that were pooled for analysis (unpaired two-tailed *t* test with Welch’s correction). Values represent means ± SEM. ns, P > 0.05; *, P ≤ 0.05; **, P ≤ 0.01; ***, P ≤ 0.001.

To investigate the promigratory effect of EEV fractions on dendritic cells in complex environments we used 3D collagen matrix migration assays, which allowed us to track the motion of individual cells (Videos 4 and 5; Sixt and Lämmermann, 2011). MMDCs were exposed to diffusion gradients of EEV-free supernatants, ss-EEV fractions, TNFα-EEV fractions, or combinatorial gradients of CCL19 and EEV-free supernatants, ss-EEV fractions, or TNFα-EEV fractions. Similar to fluorescent dextrans with a molecular weight of 30 kD, SYTO RNASelect-labeled EEVs were able to establish gradients in collagen matrices (Fig. S4, d and e). Supporting our interpretation of the transwell and the endothelial monolayer transmigration assays, gradients of EEV fractions alone had no chemotactic effect (Fig. 5, a and b; and Fig. S4 f). However, in the presence of CCL19 gradients ss-EEV fractions and TNFα-EEV fractions increased the chemotactic displacement and the chemotactic index (directionality) compared with EEV-free supernatants (Fig. 5, a and b; and Fig. S4 f).

As the directionality of migration depends critically on the sensing of extracellular guidance cues by cellular protrusions (Leithner et al., 2016), we imaged the morphology of MMDCs in the presence or absence of randomly distributed EEV fractions in a 6-µm confined microenvironment cell migration assay that lacked directional guidance cues. Compared with EEV-free supernatants, MMDCs showed increased formation of dynamic protrusions (Fig. 5, c and d; and Videos 6, 7, and 8), and correspondingly, the cells’ circularities were reduced 1.16-fold by ss-EEV fractions (P < 0.0001) and 1.4-fold by TNFα-EEV fractions (P < 0.0001; Fig. 5 e). Interestingly, the speed of migration decreased by 1.14-fold in the presence of ss-EEV fractions (P < 0.01) and 1.6-fold in the presence of TNFα-EEV fractions (P < 0.0001), whereas the migratory angle change increased by 1.42-fold (P < 0.0001) and 1.45-fold (P < 0.0001), respectively (Fig. 5 e).

In conclusion, these results indicated that LEC exosomes elicit the formation of dynamic cell protrusions that scan for extracellular guidance cues and thereby enhance directional migration of dendritic cells in complex environments. Importantly, this chemokinetic effect is independent of increasing cell migration speed but relies on the optimization of navigation.

### Induction of protrusion formation and enhancement of directional migration by TNFα-EEV fractions is dependent on GPCR signaling and CX3CL1

**Figure 6.**
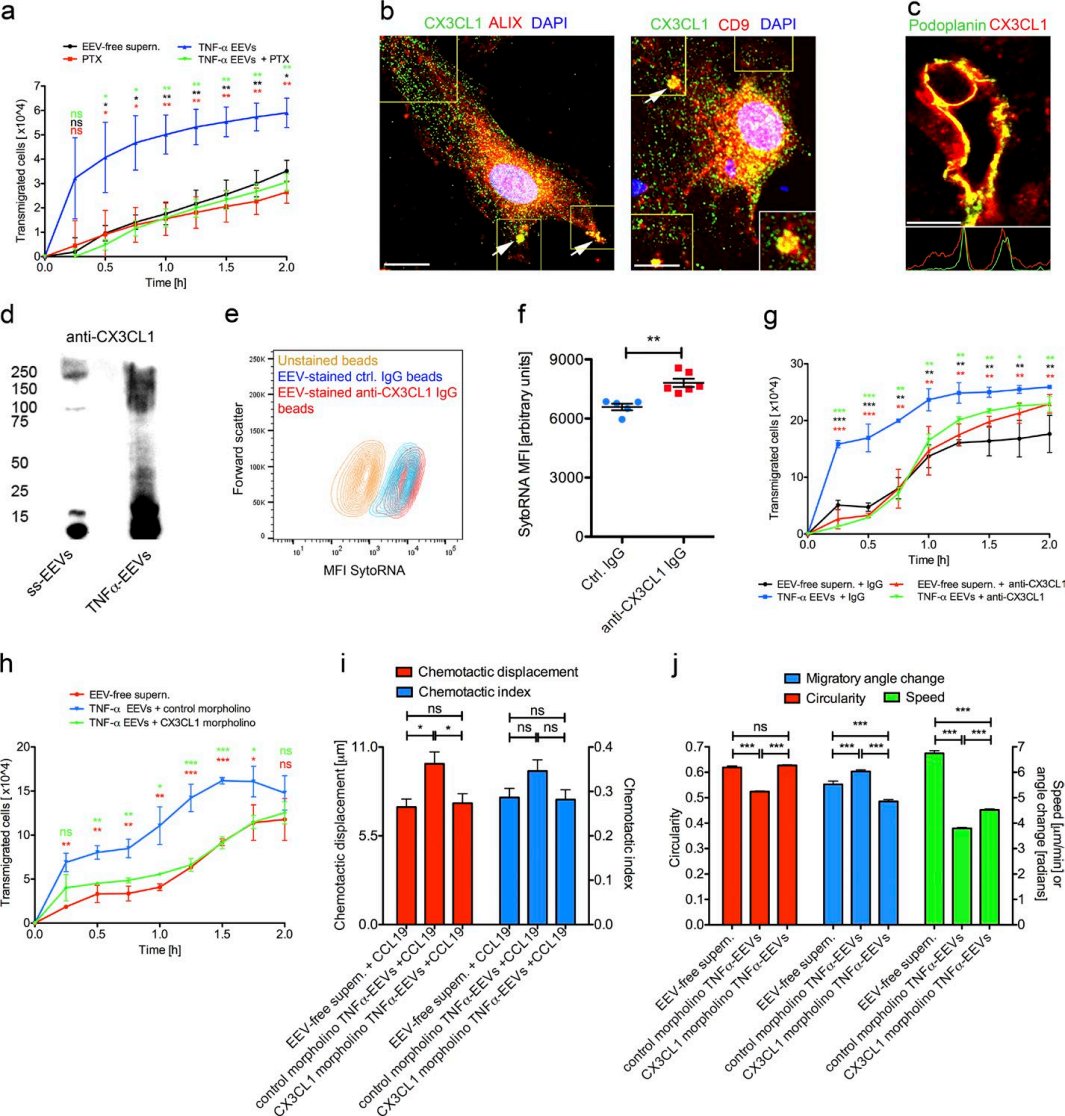
**Induction of protrusion formation and enhancement of directional migration by TNFα-EEV fractions is dependent on GPCR signaling and CX3CL1. (a)** Quantitation of transmigrated human mature MMDCs from the upper cell culture insert into the lower chamber well of a transwell assay. PTX-treated or untreated MMDCs were loaded together with EEV-free supernatants or TNFα-EEV fractions into the upper cell culture insert (*n* = 3; unpaired two-tailed *t* test). **(b)** Immunofluorescence of CX3CL1 (green) and ALIX (red; left) or CD9 (red; right) in primary human LECs. Cell nuclei are stained with DAPI (blue; *n* = 5). Yellow regions of interest were used for Manders colocalization analyses. White region of interest is a zoom. Arrows indicate colocalization of CX3CL1 with either ALIX or CD9. **(c)** Immunofluorescence of the lymphatic vessel marker podoplanin (green) and CX3CL1 (red) in human renal transplant rejections (fluorescence profiles across the respective vessels are plotted beneath the merged images; x axis: cross-sectional distance; y axis: CX3CL1- or podoplanin-specific mean immunofluorescence intensities; *n* = 6). Bars, 5 µm. **(d)** Anti-CX3CL1 immunoblot of ss-EEV fractions and TNFα-EEV fractions probed with an antibody to the C terminus of CX3CL1 (*n* = 5). Molecular masses are given in kilodaltons. **(e)** Flow cytometry contour plot of unstained beads, isotype control IgG-coated beads stained with fluorescent SYTO RNASelect-labeled TNFα-EEV fractions and anti-CX3CL1 IgG-coated beads stained with fluorescent SYTO RNASelect-labeled TNFα-EEV fractions. X axis indicates forward scatter. Y axis indicates mean fluorescence intensity (MFI) of SYTO RNASelect–labeled EEVs (*n* = 6). **(f)** Quantitation of SYTO RNASelect MFI of beads described in e (*n* = 6; unpaired two-tailed *t* test). **(g)** Quantitation of transmigrated MMDCs from the upper cell culture insert into the lower chamber well of a transwell assay. MMDCs were loaded together with EEV-free supernatants or TNFα-EEV fractions and control IgGs or anti-CX3CL1 IgGs into the upper cell culture insert (*n* = 3; unpaired two-tailed *t* test). **(h)** Quantitation of transmigrated MMDCs from the upper cell culture insert into the lower chamber well of a transwell assay. MMDCs were loaded together with EEV-free supernatants or TNFα-EEV fractions derived from 5-mispair control morpholino oligonucleotide–treated LECs or CX3CL1-specific morpholino oligonucleotide–treated LECs into the upper cell culture insert (*n* = 2; unpaired two-tailed *t* test). **(i)** Migration analyses of MMDCs in a 3D collagen matrix migration assay. Cells were exposed to gradients of EEV-free supernatants plus CCL19 (*n* = 1,070), TNFα-EEV fractions derived from 5-mispair control morpholino oligonucleotide–treated LECs plus CCL19 (*n* = 654) or TNFα-EEV fractions derived from CX3CL1-specific morpholino oligonucleotide–treated LECs plus CCL19 (*n* = 958). Red columns indicate chemotactic displacement. Blue columns indicate chemotactic index. Data are obtained from at least 218 cell tracks per experiment and three independent experiments that were pooled for analysis (unpaired two-tailed *t* test with Welch’s correction). **(j)** Migration analyses of MMDCs in a confined microenvironment migration assay in the presence of EEV-free supernatants (*n* = 1,464), TNFα-EEV fractions derived from 5-mispair control morpholino oligonucleotide–treated LECs (*n* = 9,380), or TNFα-EEV fractions derived from CX3CL1-specific morpholino oligonucleotide–treated LECs (*n* = 5,588). Red columns indicate circularity of cell shape. Blue columns indicate migratory angle change. Green columns indicate speed of migration. Data are obtained from at least 488 time points and from three independent experiments that were pooled for analysis (unpaired two-tailed *t* test with Welch’s correction). Values represent means ± SEM. ns, P > 0.05; *, P ≤ 0.05; **, P ≤ 0.01; ***, P ≤ 0.001.

Protrusion formation depends on the coupling of extracellular chemical signals to intracellular signaling pathways, which effectively translate into cellular shape change. Transmembrane signal transduction is mainly mediated by G protein–coupled receptors (GPCRs), of which many are activated by chemokines (Kufareva et al., 2015). Our proteomic analyses revealed that TNFα-EEV fractions were significantly enriched for the chemokines CCL2, CCL5, and CX3CL1 (Fig. 2 d), whose cognate GPCRs are expressed by MMDCs (Malissen et al., 2014). GPCR signaling depends on a ligand-induced conformational change in the receptor that results in the exchange of GDP for GTP on the associated G protein. In its GTP-bound form the G protein’s α subunit dissociates from the β and γsubunits and induces downstream cellular-shape-change–associated signaling cascades. To determine the involvement of GPCRs during inflammatory LEC exosome-enhanced directional dendritic cell migration, we pretreated MMDCs with pertussis toxin (PTX), which promotes the ADP-ribosylation of the G protein’s α-subunit and thereby blocks both its interaction with the GPCRs and the GTP-exchange. In the transwell migration assay PTX reduced the directional transmigration of MMDCs in response to TNFα-EEV fractions, which were added to the same upper transwell cell culture insert, by 8.35-fold (P = 0.0132) after 30 min, and 1.93-fold (P = 0.0021) after 120 min (Fig. 6 a). These results suggest that the migration-promoting effects of TNFα-EEV fractions are dependent on a GPCR signaling pathway or pathways and possibly involve one of the aforementioned and up-regulated chemokines.

Because TNFα-EEV fractions were (P = 0.02) 2.8-fold enriched for CX3CL1/fractalkine (Fig. 3 d) in comparison with ss-EEV fractions, we explored the possibility of its involvement in the promigratory effect of EEV fractions. Confocal immunofluorescence microscopy of cultured TNFα-treated primary human LECs revealed that CX3CL1/fractalkine colocalized intracellularly at the cell periphery to a high degree with the prototypical MVB and exosome marker protein CD9 (tM-CD9, 0.592 ± 0.1709; tM-CX3CL1, 0.558 ± 0.09134; Fig. 6 b) and to a lower degree with ALIX (tM-ALIX, 0.327 ± 0.214; tM-CX3CL1, 0.125 ± 0.042; Fig. 6 b). In chronically inflamed kidneys CX3CL1 immunoreactivity was found within similar perilymphatic halos (Fig. 6 c) that were positive for CD9 and CD63 (Fig. 1, a–c), and CX3CR1^+^ cells were found to associate with and intravasate lymphatic vessels (Fig. S5 a). Immunoblotting with antibodies specific for the C-terminal region of CX3CL1 (Fig. S5 b) confirmed up-regulation of the full-length transmembrane form of CX3CL1 in EEV fractions upon TNFα stimulation (Fig. 6 d). Flow cytometry of anti-CX3CL1 IgG-coated beads revealed a 19% (P = 0.0017) higher binding capacity for TNFα-EEVs than beads that were coated with unspecific control IgG (Fig. 6, e and f). These results indicated that CX3CL1, through its location in perivascular halos and its presentation at the surface of LEC exosomes, is a plausible candidate to mediate the promigratory EEV effects.

Because CX3CL1 also has been shown to promote migration of dendritic cells toward afferent lymphatics (Johnson and Jackson, 2013), we sought to address whether this chemokine was responsible for the chemokinetic enhancement of directional MMDC migration in response to TNFα-EEV fractions. Importantly, MMDCs stained positively for CX3CR1 in flow cytometry (Fig. S5 c). Simultaneous application of TNFα-EEV fractions and isotype control IgGs to the upper cell culture insert of transwell migration assays increased directional transmigration of MMDCs by 3.58-fold (P < 0.0001) in the initial 30 min and 1.47-fold (P < 0.001) after 120 min compared with EEV-free supernatants, whereas the presence of neutralizing anti-CX3CL1 monoclonal IgGs (mIgGs) inhibited the promigratory effect of TNFα-EEV fractions (Fig. 6 g). These findings were further corroborated by the use of CX3CL1 knockdown EEV fractions (Fig. S5, d–f). Whereas mispair control knockdown ss-EEV fractions (Fig. S5 g) and TNFα-EEV fractions (Fig. 6 h) loaded into the upper cell culture inserts enhanced directional transmigration of MMDCs in the transwell migration assay, CX3CL1 knockdown ss-EEV fractions (Fig. S5 g) and TNFα-EEV fractions (Fig. 6 h) had little or no effect when compared with EEV-free supernatants. Similarly, in the complex environment of a 3D collagen matrix migration assay the combinatorial gradients of CCL19 and CX3CL1 knockdown TNFα-EEV fractions had no promigratory effect on MMDCs, whereas combinatorial gradients with mispair control knockdown TNFα-EEV fractions enhanced the chemotactic displacement by 1.37-fold, when compared with CCL19 gradients alone (P ≤ 0.05, Fig. 6 i). Indeed, in confined microenvironment migration assays CX3CL1 knockdown TNFα-EEV fractions, similar to EEV-free supernatants and in contrast with mispair control knockdown TNFα-EEV fractions, were unable to induce protrusion formation (Fig. 6 j). These results suggest that inflammatory LEC exosomes in perivascular halos use the CX3CL1–CX3CR1 signaling axis to promote directional migration of MMDCs through complex environments by inducing the formation of dynamic cellular protrusions that probe the extracellular space for guidance cues.

### CX3CL1 in EEV fractions promotes transmigration of prostate carcinoma cells

**Figure 7.**
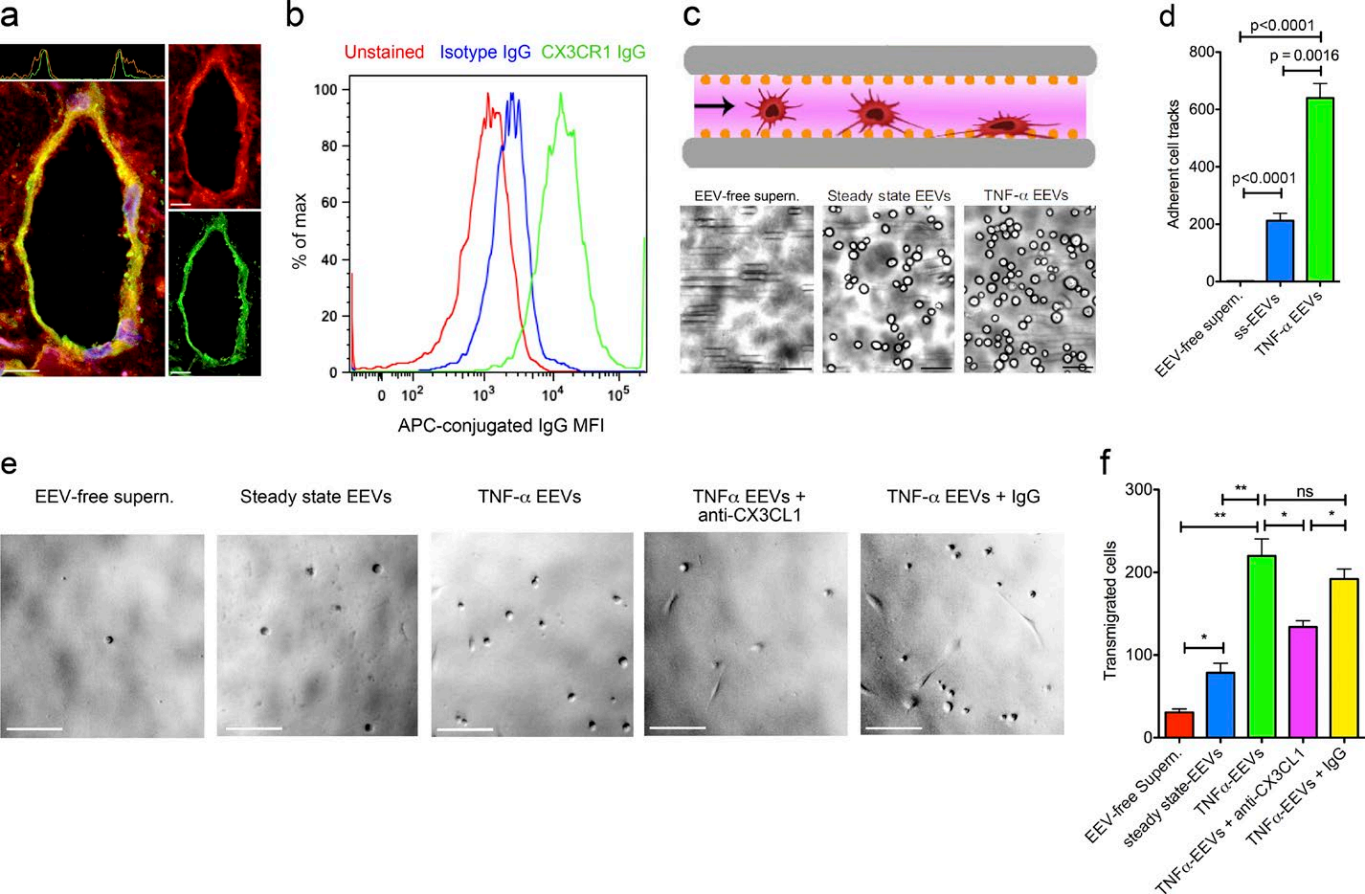
**CX3CL1 in EEV fractions promotes transmigration of prostate carcinoma cells. (a)** Immunofluorescence of CD9 (red) and podoplanin (green) in the peritumoral stroma of a human prostate carcinoma (fluorescence profiles across respective vessels are plotted above the merged images; x axis, cross-sectional distance; y axis, CD9- or podoplanin-specific mean immunofluorescence intensities; *n* = 5). Bars, 10 µm. **(b)** Flow cytometry histogram of unstained (red), APC-conjugated isotype IgG-stained (blue), and APC-conjugated anti-CX3CR1 IgG-stained (green) PC3-ML prostate carcinoma cells. X axis, percentage of maximum; Y axis, mean fluorescence intensity (MFI) of APC-conjugated IgG (*n* = 4). **(c)** Microfluidic adhesion setup (top). EEV fractions were immobilized in microfluidic capillaries, and the adhesions of PC3-ML prostate carcinoma cells were acquired under constant-flow conditions for 2 min. Brightfield images of cancer cells (bottom) that adhered to EEV-free supernatant-coated (left), ss-EEV fraction-coated (middle), or TNFα-EEV fraction-coated (right) microfluidic channels (*n* = 3). Bars, 50 µm. **(d)** Automated quantitation of adherent PC3-ML cell tracks in EEV-free supernatant-coated, ss-EEV fraction-coated, or TNFα-EEV fraction-coated (*n* = 3; unpaired two-tailed *t* test) microfluidic channels. **(e)** Brightfield images of transmigrated PC3-ML prostate carcinoma cells from the upper cell culture insert into the lower chamber well of a transwell assay. PC3-ML cells were loaded into the upper cell culture inserts after EEV-free supernatants (leftmost), ss-EEV fractions (center left), TNFα-EEV fractions (middle), TNFα-EEV fractions plus anti-CX3CL1 IgG (center right), or TNFα-EEV fractions plus isotype control IgG (rightmost) were loaded into the bottom chamber wells (*n* = 3). Bars, 100 µm. **(f)** Quantitation of transmigrated PC3-ML cells described in d (*n* = 3; unpaired two-tailed *t* test). ns, P > 0.05; *, P ≤ 0.05; **, P ≤ 0.01.

To determine whether LEC exosomes might also promote directional migration of nonhematopoietic cells through complex environments in distinct pathophysiological settings, we immunostained human prostatectomy specimens and found that, similarly to chronically inflamed tissues, CD9^+^ areas formed around lymphatic vessels in the peritumoral stroma (Fig. 7 a). Prostate carcinomas frequently invade lymphatic vessels and form metastases in a CX3CR1-dependent manner (Shulby et al., 2004; Andre et al., 2006; Marchesi et al., 2008; Castellana et al., 2009; Locatelli et al., 2010; Yao et al., 2014; Shen et al., 2016). In a microfluidic capillary adhesion assay the widely used PC3-ML prostate carcinoma cell line, which expresses CX3CR1 (Fig. 7 b; Shulby et al., 2004), bound to immobilized ss-EEV and TNFα-EEV fractions (Fig. 7, c and d). Similar to the results obtained with MMDCs, ss-EEV fractions and TNFα-EEV fractions but not EEV-free supernatant increased the directional transwell transmigration of tumor cells by 2.59-fold and 7.26-fold, respectively (Fig. 7, e and f). Importantly, the promigratory effects of TNFα-EEV fractions were blocked by neutralizing anti-CX3CL1 mIgGs (P = 0.05), whereas unspecific control IgGs had no significant effect (Fig. 7, e and f). These findings indicate that LEC exosomes can promote CX3CL1-mediated directional migration across different cell types.

## Discussion

On their journey to the nearest draining lymph node, migratory dendritic cells and cancer cells have to pass through complex and varying microenvironments. LECs secrete CCL21 to form chemotactic and haptotactic gradients for the attraction of dendritic cells into the lymphatic vessel lumen (Johnson and Jackson, 2010; Schumann et al., 2010; Weber et al., 2013; Vaahtomeri et al., 2017). In this study, we describe previously unrecognized halos in the perilymphatic stroma of inflamed tissues, distinguished by their expression of the exosome marker proteins CD9 and CD63. Furthermore, vesicles with sizes and buoyant densities consistent with those of exosomes and expressing the same exosomal marker proteins were abundantly enriched in primary human LEC-derived basolateral TNFα-EEV fractions. Comprehensive quantitative proteomic analyses yielded an inventory of 1,717 proteins in EEV fractions, of which 86% were up-regulated by at least 1.5-fold upon TNFα stimulation. Importantly, the general degree of protein up-regulation correlated with the increase in vesicle numbers in TNFα-EEV fractions and suggested that the mean protein load and composition per vesicle was relatively similar to that of ss-EEV fractions. Among the proteins that were enriched above average in TNFα-EEV fractions were well-characterized cell migration–promoting proteins including growth factors, cytokines, chemokines, cytoskeletal (regulatory) proteins, motor proteins, adhesion proteins, and proteolytic enzymes. Moreover, TNFα-EEV fractions were highly enriched for inflammation-associated proteins, antigen presentation, and processing and proteasomal proteins, which have recently been linked to the immunomodulatory properties of exosome-like vesicles (Walker et al., 2009; Dieudé et al., 2015). In contrast, several proteins, notably ECM proteins and matrix-remodeling enzymes were less enriched in TNFα-EEV fractions.

The topographical localization of exosome markers in inflamed tissues and the motility-promoting protein cargo of EEV fractions raised the question of whether exosomes might influence the migration of cells toward and into lymphatic vessels. We were able to show that EEV fractions are taken up by MMDCs. The presence of randomly distributed TNFα-EEV fractions significantly enhanced the ex vivo directional migration of MMDCs toward lymphatic vessels in tissue explants and the in vitro directional transmigration of MMDCs through CCL21-overexpressing primary human LEC monolayers. Single-cell tracking analyses in 3D collagen matrix migration assays revealed that gradients of TNFα-EEV fractions were not chemotactic as such but rather enhanced the displacement of MMDCs along chemotactic cues (CCL19 gradients) by raising their directionality (chemotactic index) through chemokinesis. We have recently shown that the extent of cellular protrusions such as filopodia and lamellipodia positively impacts the ability of dendritic cells to sense and respond to directional guidance cues (Leithner et al., 2016), although it negatively correlates with the speed of migration. In this study, we found that exposure of MMDCs to EEV fractions in confined microenvironments that lacked directional guidance cues caused a strong increase of dynamic cellular protrusion formation and induced frequent directional changes while reducing cell migration speed. These results suggest that EEV fractions promote directional migration in complex environments by improving navigation, which is independent of increasing cellular migration speed, but relies on the induction of exploratory dynamic cell protrusions.

These results were corroborated in the reductionist transwell migration assay, where MMDCs were challenged to migrate along geometric guidance cues of a porous maze. Compared with EEV-free supernatant, EEV fractions increased the directional transmigration of MMDCs, regardless of whether the EEV fractions were added to the lower chamber or were mixed with the cells in the upper cell culture insert, consistent with their ability to promote such motility through chemokinesis.

PTX pretreatment of MMDCs revealed that EEV fraction-mediated enhancement of directional transwell transmigration was dependent on signaling via GPCRs, and intriguingly, our proteomic profiles indicated the presence of several of their chemokine ligands, including CX3CL1. We have reported previously that the cleaved soluble ectodomains of transmembrane CX3CL1/fractalkine strongly enhance the in vitro and in vivo lymphatic transmigration of mature dendritic cells via CX3CR1, the only known receptor for CX3CL1 (Johnson and Jackson, 2013). These results are consistent with studies that mice with genetic CX3CL1 or CX3CR1 deficiency show severe migration defects of inflammatory cells in the skin, gut, and kidney (Niess et al., 2005; Furuichi et al., 2006; Ishida et al., 2008; Boivin et al., 2012). CX3CL1 colocalized intracellularly with MVB and exosome markers in TNFα-stimulated LECs, and similar to CD9 and CD63, we found its presence in halos surrounding lymphatic vessels of chronically inflamed kidney transplants. The full-length form of CX3CL1 that includes the transmembrane anchor was expressed on the surface of EEVs and was up-regulated in TNFα-EEV fractions. Importantly, in transwell assays, CX3CL1 neutralizing mIgGs (Held-Feindt et al., 2010; Johnson and Jackson, 2013) as well as CX3CL1 knockdown by using morpholino oligonucleotides completely abolished the migration-promoting effect of TNFα-EEV fractions on CX3CR1^+^ MMDCs. Moreover, knockdown of CX3CL1 in TNFα-EEV fractions inhibited the enhanced chemotaxis of MMDCs toward CCL19 gradients and the induction of dynamic cell protrusion formation of MMDCs in the absence of directional guidance cues.

Prostate cancers have a high propensity to metastasize into regional lymph nodes, and their expression of CX3CR1 correlates positively with the metastatic proclivity (Shulby et al., 2004; Andre et al., 2006; Marchesi et al., 2008; Castellana et al., 2009; Locatelli et al., 2010; Yao et al., 2014; Shen et al., 2016). In this study, we have shown that CD9^+^ halos surround lymphatic vessels in human prostate cancer specimens and that EEV fractions promoted the migration of CX3CR1^+^ human PC3-ML prostate cancer cells in a CX3CL1-dependent manner. These results provide a first indication that perilymphatic LEC exosomes can promote directional migratory responses across different cell types via CX3CL1.

Collectively, this study introduces the novel concept of a basolateral promigratory layer of LEC-derived exosomes surrounding lymphatic vessels in inflammation and cancer. We conclude that inflammatory LEC exosomes, in a chemokinetic GPCR-signaling–dependent manner, induce the formation of dynamic exploratory cell protrusions via membrane bound CX3CL1 and thereby increase the directional movement of CX3CR1^+^ cells along guidance cues in complex environments. However, the contribution of additional exosome-induced migration machinery activating mechanisms cannot be ruled out. Beyond these findings the present study provides a comprehensive quantitative analysis of the composition of ss and inflammatory LEC exosomes with a large inventory of proteins whose potential relevancies in health and disease remain to be explored.

## Materials and methods

### Human and mouse tissues

The use of human tissue samples was approved by the Ethical Committee of the Medical University of Vienna (approval EK-Nr 270/2006) in compliance with Austrian legislation. Samples of formaldehyde-fixed, paraffin-embedded residual tissues were retrieved form the archive of the Clinical Institute of Pathology of the Medical University of Vienna. Animal procedures were approved by the Animal Experimental Committee of the Medical University of Vienna and by the Austrian Ministry of Science (license number 66.018/0027-WF/V/3b/2014). M. Detmar (ETH Zurich, Zurich, Switzerland) provided formaldehyde-fixed, paraffin-embedded mouse ears with 0, 7, and 21 d of experimental oxazolone-induced dermatitis (Zgraggen et al., 2014).

### LEC culture

Primary human dermal microvascular LECs (C12260; PromoCell) were expanded in endothelial cell growth medium-2 (EGM2MV; Lonza) until passage three, enriched for podoplanin^+^ cells by flow cytometry, further expanded once and then stored in liquid N_2_. For all experiments cells were used in passage five.

### Harvesting basolateral culture supernatant from LEC transwell cultures

Early passage LECs were cultured to confluence in polycarbonate six-well transwell cell culture inserts (0.4-µm pore size, 10^8^ pores/cm^2^; Corning) in EGM2MV media. Leakage was tested on monolayers with 70-kD FITC-dextran (10 µg/ml; Sigma-Aldrich). Maximal permeability was determined without cells. Basolateral LEC culture supernatants were collected after 24 h of culturing in EGM2MV media that was supplemented with 10% exosome-free FBS (Thermo Fisher Scientific) instead of 10% FBS, which is contaminated by bovine-derived exosomes as provided by the manufacturer. In some experiments LECs were incubated in exosome-free EGM2MV media containing 7 ng/ml TNF-α (R&D Systems).

### Isolation of EEV fractions

EEV fractions were isolated with the ExoQuick-TC exosome precipitation solution (System Biosciences) by following the manufacturers protocol. In brief, basolateral LEC culture supernatants were centrifuged at 1,500 *g* and filtered through a 0.2-µm filter (Falcon) to remove cellular debris and larger vesicles, followed by incubation with ExoQuick-TC solution for 12 h at 4°C and precipitation of exosomal vesicles by centrifugation at 1,500 *g*. Protein content was determined with the BCA protein assay kit (Thermo Fisher Scientific) by following the manufacturers protocol and expressed as mg protein per ml medium.

### Immunoelectron microscopy

EEV fractions were fixed in 2% PFA, deposited on formvar carbon-coated EM nickel grids, and allowed to adsorb for 20 min in a dry environment. Afterward the grids were washed in drops of PBS and fixed in drops of 1% glutaraldehyde. Samples were contrasted in a solution of uranyl oxalate, pH 7.0, and covered with a polyvinyl alcohol film. Some grids were labeled with mouse CD63 (Santa Cruz Biotechnology, Inc.) and detected with 15 nm immunogold conjugate EM.GAM15 (BBInternational) as described (Théry et al., 2006). The grids were observed under a JEM-1010 (JEOL USA) at 60 KV. The EEVs were semiautomatically segmented with Ilastik imaging software (Kreshuk et al., 2011), and diameters of binary images were calculated with the particle analyzer of Fiji imaging software (ImageJ; National Institutes of Health; Schindelin et al., 2012). Immunogold EM of human dermis and kidneys was performed on Lowicryl-K4M embedded sections as described previously (Kerjaschki et al., 2004).

### Confocal immunofluorescence microscopy

Antibodies to mouse podoplanin were homemade (Breiteneder-Geleff et al., 1999); antibodies to CD31 were purchased from R&D Systems; and antibodies to CD9, CD63, CX3CR1, and CX3CL1 were from Santa Cruz Biotechnology, Inc. or Abcam. Immunofluorescence staining was performed on 4-µm paraffin sections of kidneys from renal transplant rejection (*n* = 6), synovias of rheumatoid arthritis (*n* = 4), intestines of Crohn’s disease (*n* = 4), skins of atopic dermatitis (*n* = 3), and skins of oxazolone-induced hypersensitivity dermatitis (*n* = 3) as described previously (Kerjaschki et al., 2004).

Secondary antibodies (Alexa Fluor 488, Alexa Fluor 594, or Alexa Fluor 633) were purchased from Molecular Probes. Images were acquired on an inverted confocal LSM700 (Zeiss) microscope with a 20× 0.8 NA air Plan Apochromat objective (ZEISS) for perivascular localizations of proteins or a 63× 1.4 NA oil Plan Apochromat objective (ZEISS) for intracellular localizations of proteins. Zen 2012 software (ZEISS) was used for image acquisitions. The perivascular distribution of CD9-associated immunofluorescence was plotted with the Fiji imaging software (Schindelin et al., 2012), and the relative fluorescence diameters were calculated.

### Gel electrophoresis, Coomassie blue staining, and immunoblotting

Sodium dodecyl sulfate PAGE, Coomassie blue staining, and immunoblotting were performed as described previously (Maurer et al., 2013). The antibodies against human β-actin, CD9, CD63, and CX3CL1 were purchased from Santa Cruz Biotechnology, Inc.

### OptiPrep density gradient centrifugation

The procedure was performed as previously described (Lobb et al., 2015) by using the OptiPrep (Sigma-Aldrich), a 70% solution of iodixanol in water with a density of 1.32 g/ml. A discontinuous iodixanol gradient was prepared by diluting the stock solution with 0.25 M sucrose/10 mM Tris, pH 7.5 (Sigma-Aldrich) to generate 40, 20, 10, and 5%. The solutions were layered into 12-ml ultracentrifuge tubes with EEV fractions on top of the 5% layer. The discontinuous density gradient was centrifuged by using a P40ST rotor (Hitachi) for 16 h at 100,000 *g* at 4**°**C. 10 fractions were collected from the top of the gradient, weighed, and analyzed by gel electrophoresis, Coomassie blue staining, and immunoblotting with anti-CD63 (Santa Cruz Biotechnology, Inc.) antibody.

### NTAs

NTAs were performed as recently described (Vestad et al., 2017). In brief, the size and concentration of the EEV fractions were analyzed by using the NanoSight NS-500 system (Malvern Instruments). Each EEV fraction was diluted in exosomes-free prefiltered PBS (Gibco) and vortexed for 1 min to obtain measurable concentrations between 0.5 × 10^8^ and 5 × 10^9^ particles/ml. For analyses, a monochromatic laser beam (532 nm) was applied to the diluted exosomes. NTA software version 3.1 analyzed the samples at a constant temperature (22°C). As a control 100-nm polystyrene latex microspheres (Duke Scientific) were used. The NTA software produced five videos of 30-s duration, with a 5-s delay between the recordings creating five replicate histograms that were averaged to give the final estimate of the particle sizes and concentrations. NTA settings were preoptimized and kept constant between samples.

### Filter-aided sample preparation of EEV fractions and TMT-based LC-MSMS analysis

Freshly prepared EEV fractions were resuspended in filter-aided sample preparation lysis buffer (50 mM Hepes [Sigma-Aldrich], pH 8.0; 4% SDS [Sigma-Aldrich]; protease inhibitor cocktail [cOmplete; Roche]) and incubated at 99°C for 5 min. The EEV fraction lysates were sonicated in microTUBES (duty cycle 10, intensity 5, cycle number 200, duration of 80 s, and a power of 26 W; Covaris). After centrifugation at 16,000 *g* for 10 min at 20°C the protein concentrations of the lysis supernatants were determined with the BCA Protein Assay (Thermo Fisher Scientific) and 50 µg protein per sample was reduced with 100 mM DTT (Sigma-Aldrich) at 99°C for 5 min. The filter-aided sample preparation was performed essentially as described previously (Manza et al., 2005; Wiśniewski et al., 2009; Maurer et al., 2013). In brief, protein lysates were loaded onto 30-kD filter units (Microcon) and washed extensively with 8 M urea (Sigma-Aldrich) and 50 mM triethylammonium bicarbonate buffer (TEAB; Sigma-Aldrich) to completely remove SDS. Upon alkylation with iodoacetamide (Sigma-Aldrich) and further wash steps with TEAB, proteins were digested with 0.625 µg trypsin (Promega) in 50 mM TEAB, pH 8.5, overnight at 37°C and sequentially eluted with 50 mM TEAB and 0.5 M NaCl. Tryptic peptides were desalted by solid phase extraction with C18 sorbent material (Empore). Bound peptides were washed with a buffer containing 0.4% formic acid (Sigma-Aldrich) and 2% trifluoracetic acid (Sigma-Aldrich) and were eluted with 90% acetonitrile.

The derivatization of trypsin-digested samples with 6-plex TMTs was performed according to the instructions supplied by the manufacturer (Thermo Fisher Scientific). The samples were then pooled in ratios calculated such that the total intensity of the reporter ion signals was equal for abundant proteins. The pooled peptides were fractionated into 20 fractions by reverse-phase liquid chromatography as described previously (Maurer et al., 2013).

LC-MSMS was performed on a hybrid linear trap quadrupole Orbitrap Velos mass spectrometer (Thermo Fisher Scientific). The analyses were performed with the top 10 high-energy collision-induced dissociation method for peptide identification plus relative quantitation of TMT reporter ions as previously described (Maurer et al., 2013).

#### LC-MSMS data analysis, TMT quantitation, and Top3 quantitation

The acquired raw MS data files were processed as previously described (Maurer et al., 2013). The resultant peak lists were searched against the human SwissProt database version 20150601 with the search engines Mascot (v.2.3.02) and Phenyx (v.2.5.14).

For TMT quantitation, the isobar R package was used (Breitwieser et al., 2011). The TMT reporter ion intensities were normalized in silico to give an equal median intensity in each TMT reporter channel and then multiplied by the measured protein concentration of each input sample and divided by the minimal protein concentration of the six samples. In this way the reporter ion intensities are proportional to the measured protein concentrations. A protein is considered significantly regulated if both p-value sample and false discovery rate–adjusted p-value ratio from the isobar model are <0.05.

The interprotein abundance of individual proteins was calculated by using the Top3 quantitation method (Silva et al., 2006).

### Mean interprotein abundances of biologically relevant protein clusters

The proteomic results were analyzed by using the bioinformatic platform DAVID (Huang da et al., 2009) to search the Gene Ontology database and the extracellular vesicle–specific databases ExoCarta (Keerthikumar et al., 2016) and EVpedia (Kim et al., 2015). The mean interprotein abundances of clusters were calculated from all proteins that comprise a respective cluster.

### Preparation of primary human mature MMDCs

MMDCs were generated as described previously (Bruckner et al., 2012). In brief, mononuclear cells were isolated from whole blood of human male volunteers between 20 and 30 yr of age by using Ficoll (Miltenyi Biotec) gradient centrifugation and enrichment with anti-CD14 coated MACS beads (Miltenyi Biotec) by following the manufacturer’s instructions. The mononuclear cells were differentiated into immature dendritic cells by culturing in 1 ml RPMI (Gibco) containing 10% FBS (R10), 50 ng IL-4 (PeproTech), and 50 ng granulocyte macrophage colony-stimulating factor (PeproTech) for 6 d in a 24-well format (0.5 ml fresh media was added every 2 d of culture). For maturation, 10^6^ cells were cultured in R10 media containing 50 ng/ml IL-4, 50 ng/ml granulocyte macrophage colony-stimulating factor, 10 µg/ml prostaglandin E2 (Sigma-Aldrich), 20 ng/ml TNFα (R&D Systems), 15 ng/ml IL-6 (PeproTech), and 20 ng/ml IL-1β (PeproTech) for 48 h or with 1 µg/ml lipopolysaccharide (from *Salmonella abortus*; Sigma-Aldrich) for 24 h.

### Uptake of EEVs by MMDCs

EEV fractions from 2.6 ml basolateral LEC culture supernatant were labeled per sample with the cell-permeant lipophilic membrane-specific PKH-67 dye (Sigma-Aldrich) or the RNA-specific SYTO RNASelect green fluorescent dye (Invitrogen), and excess dyes were removed with Exosome spin columns (Invitrogen) by following the manufacturer’s instructions. An equally sized volume of EEV-free basolateral culture supernatant was used as a control. Uptake of PKH-67-stained EEV fractions or EEV-free supernatants by MMDCs was determined by incubation at 4°C and 37°C for 60 min followed by flow cytometry. The fluorescence intensities of the cells were plotted with the FlowJo software (BD), and the mean fluorescence intensity of MMDCs was calculated.

For the uptake of SYTO RNASelect–stained EEV fractions, MMDCs were stained with NucBlue live cell stain (Molecular Probes) and resuspended at a concentration of 2 × 10^6^ cells/ml R10. 100 µl collagen polymerization solution (30 µl 10× MEM [Gibco], 15 µl sodium bicarbonate buffer, and 225 µl PureCol bovine collagen I [Advanced BioMatric]) was mixed with 50 µl MMDCs, transferred into custom-built collagen chambers, and incubated for polymerization at 37°C for 60 min. EEV fractions were transferred into the collagen chambers to form soluble gradients, and cells were fixed with 4% PFA after 3 min. Fluorescent images were acquired on an inverted confocal LSM700 microscope with 40× 1.2 NA water Plan Apochromat objective using the 2012 software (ZEISS).

### Transwell migration assay with MMDCs

EEV fractions from 4.7 ml basolateral LEC culture supernatant were resuspended in 10 µl R10 per sample. An equally sized volume of EEV-free basolateral culture supernatant was used as a control. For fluorescence labeling, MMDCs were incubated with 2.5 µM cell tracker green (5-chloromethylfluorescein diacetate; CMFDA; Invitrogen) for 40 min, washed in R10, and then allowed to recover for 30 min. EEV fractions or EEV-free supernatants were applied to either the lower chamber wells or upper Fluoroblok cell culture inserts (3-µm pore size, 8 × 10^5^ pores/cm^2^; BD) before the addition of fluorescently labeled MMDCs to the upper Fluoroblok cell culture inserts. To investigate the involvement of CX3CL1 in EEV fraction-promoted MMDC migration, rabbit anti–human CX3CL1-neutralizing IgGs (AMS Biotechnology) or isotype-control IgGs (R&D Systems) were applied to the upper Fluoroblok cell culture insert at the same time as EEV fractions or EEV-free supernatants. Migration of MMDCs through the 3-µm pores of the 10-µm-thick light-opaque polyethylene membrane into the lower chamber well was measured every 15 min on an automated fluorescent plate reader (Synergy HT; BioTek) at 37°C (Johnson et al., 2006). The fluorescence signal was calibrated against a standard curve, and migration was expressed as the number of transmigrated MMDCs in the lower chamber. The mean kinetic curves of transmigrated MMDCs were calculated.

The contribution of GPCRs was measured by preincubating cell tracker–stained MMDCs for 2 h with PTX (200 ng/ml; EMD Millipore) in the presence of 25 mM Hepes, pH 7.4 (Sigma-Aldrich). The cells were then washed in R10 and applied to the upper Fluoroblok cell culture insert at the same time as EEV fractions or EEV-free supernatants.

### Endothelial monolayer transmigration assay

The assay was performed as described by Vaahtomeri et al. (2017). In brief, EEV fractions were isolated from 3.1 ml basolateral LEC culture supernatant and resuspended in 10 µl EGM2MV per sample. An equally sized volume of EEV-free basolateral LEC culture supernatant was used as a control. Eight-well glass bottom slides (Ibidi) coated with 0.1% gelatin (Sigma-Aldrich) were seeded with LECs and cultured in EGM2MV. When LECs reached a confluence of 50%, they were transduced with a lentiviral construct encoding mouse CCL21 (Russo et al., 2016). The media were changed after 20 h and subsequently every 48 h after that until cells reached 100% confluence. 5 × 10^6^ MMDCs were fluorescently labeled for 15 min at room temperature in the dark with 6.7 µM 5-carboxytetramethylrhodamine (Thermo Fisher Scientific) in 1 ml R10. After washing, MMDCs were resuspended at a density of 4 × 10^5^ cells/ml EGM2MV and allowed to recover for 40 min. 10^5^ MMDCs were transferred onto the endothelial monolayer, and glass slides were mounted onto an inverted widefield Eclipse (Nikon) microscope equipped with an incubator at 37°C and 5% CO_2_. Upon addition of EEV fractions or EEV-free supernatants, brightfield and fluorescence images were acquired every 2.2 s for 40 min with NIS Elements AR 4.00.08 software (Nikon). The number of transmigrated cells was determined manually.

### Ex vivo split-ear crawl-in migration assay

EEV fractions were isolated from 2.6 ml basolateral LEC culture supernatant and resuspended in 10 µl R10 per sample. An equally sized volume of EEV-free basolateral culture supernatant was used as a control. BALB/c Prox1-GFP reporter mice (Choi et al., 2011) were killed by cervical dislocation, and their ears were excised postmortem. The ears were split in half, and the fat-free ventral side was stretched and mounted flat onto an R10-filled holder, with the lymphatic vasculature facing upward (Weber and Sixt, 2013). 5 × 10^6^ MMDCs were fluorescently labeled for 15 min at room temperature in the dark with 6.7 µM 5-carboxytetramethylrhodamine (Thermo Fisher Scientific) in 1 ml R10. After washing, cells were resuspended at a concentration of 3 × 10^5^ cells per ml R10 containing EEV fractions or EEV-free supernatants and were immediately transferred onto mounted ventral ear sides. Cells were allowed to migrate for 3 h at 37°C and 5% CO_2_ before tissues were fixed with 4% PFA and mounted for imaging on an inverted confocal LSM700 microscope with a 20× 0.8 NA Air Plan Apochromat objective. Fluorescence image stacks of the entire ear were acquired with 6-µm step sizes with the Zen 2012 software. Semiautomatic segmentation and minimum distance measurements between MMDCs and the nearest Prox1-GFP–positive lymphatic vessels in fat-free areas were performed with Imaris imaging software (Bitplane) and normalized to the overall vessel density.

### 3D collagen matrix migration assay

EEV fractions were isolated from 2.6 ml basolateral LEC culture supernatant and resuspended in 10 µl R10 per sample. An equally sized volume of EEV-free basolateral LEC culture supernatant was used as a control. MMDCs were resuspended at a concentration of 2 × 10^6^ cells per ml R10. 100 µl collagen polymerization solution (30 µl 10× MEM [Gibco], 15 µl sodium bicarbonate buffer, and 225 µl PureCol bovine collagen I [Advanced BioMatrix]) were mixed with 50 µl MMDCs, transferred into a custom-built migration chamber, and incubated for polymerization at 37°C for 1 h. The gradient solution was prepared with 10 µl EEV fractions or EEV-free supernatants, 8 µl R10, and 2 µl human CCL19 (100 ng/ml; PeproTech) and was transferred into the migration chamber to form a soluble chemotactic gradient. Brightfield images were acquired with inverted DM IL light-emitting diode microscopes (Leica Microsystems) with 4× 0.10 NA HI Plan objectives (Leica Microsystems) and the SV-Timelapse software (SVS-Vistek) every 60 s at 37°C and 5% CO_2_ for 6 h. Semiautomatic segmentation and migration analysis of MMDCs were performed with the autoregressive tracking mode of Imaris imaging software. Only cells with a minimum track displacement length of 24.1 µm were considered for analyses.

### Confined microenvironment migration assay

EEV fractions were isolated from 2.6 ml basolateral LEC culture supernatant and resuspended in 10 µl R10 per sample. An equally sized volume of EEV-free basolateral LEC culture supernatant was used as a control. MMDCs were resuspended at a concentration of 10^8^ cells/ml R10. Confiners with 6-µm spacer pillars were molded in polydimethylsiloxane (Sylgard; Sigma-Aldrich) from photolithographically developed masters as previously described (Leithner et al., 2016). 10 µl EEV fractions or EEV-free supernatant was mixed with 20 µl cell suspension and dropped onto the confiner before magnetic mounting onto glass plates. Brightfield images were acquired with inverted DM IL light-emitting diode microscopes with 10× 0.22 NA HI Plan objectives (Leica Microsystems) and the SV-Timelapse software every 60 s at 37°C and 5% CO_2_ for 3 h. Semiautomatic segmentation was performed with Ilastik imaging software (Kreshuk et al., 2011), semiautomatic migration analyses of MMDCs were performed with Imaris imaging software, and morphometric measurements were performed with Fiji imaging software (Schindelin et al., 2012). Only migrating cells were considered for analyses.

### CX3CR1 immunolabeling and flow cytometry of MMDCs and PC3-ML prostate carcinoma cells

PC3-ML human prostate carcinoma cells were grown in DMEM (+10% FBS; Gibco) to 80% confluency and were then trypsinized, washed, and resuspended at a concentration of 10^6^ cells/ml MACS flow cytometry buffer (Miltenyi Biotec). MMDCs were resuspended at a concentration of 10^6^ cells/ml MACS flow cytometry buffer. 10^5^ cells were stained with allophycocyanin (APC)-conjugated rat anti–human CX3CR1 IgG (BioLegend) or rat isotype control IgG (BioLegend) for 60 min at 4°C. Cells were then washed with and resuspended in MACS flow cytometry buffer and subjected to flow cytometry on a FACS ARIA III (BD). The data were analyzed with the FlowJo software.

### Flow cytometry of isotype IgG- and anti-CX3CL1 mIgG-coated *N*-hydroxysuccinimide beads incubated with SYTO RNASelect–labeled EEV fractions

EEV fractions were isolated from 5 ml basolateral LEC culture supernatant, stained with RNA-specific SYTO RNASelect green fluorescent dye (Invitrogen) and resuspended in 45 µl PBS per sample. PureProteome *N*-hydroxysuccinimide FlexiBind magnetic beads (EMD Millipore) were coated with IgGs according to the manufacturer’s instructions. In brief, 25 µl bead slurry was freed from storage buffer, and beads were equilibrated with 1 mM HCl in PBS. IgGs were resuspended at a concentration of 0.5 mg/ml in PBS. Beads were resuspended in 45 µl IgG solutions and incubated for 2 h at room temperature on an orbital shaker. The beads were washed and incubated for 1 h in quench buffer (100 mM Tris-HCl and 150 mM NaCl, pH 8.0) and after washing with PBS were resuspended in 45 µl fluorescently labeled EEV fractions. After 15 min of incubation at room temperature, beads were washed with PBS and subjected to flow cytometry on a FACS ARIA III. The data were analyzed with the FlowJo software.

### Mispair control CX3CL1 or CX3CL1-specific morpholino oligonucleotide knockdown of LECs

LECs were cultured to 80% confluency in transwell cell culture inserts in EGM2MV (Lonza) media as described above. LECs were then cultured for additional 72 h to 100% confluency in the presence of 10 µM 5-mispair control CX3CL1 morpholino oligonucleotides (5′-TgCCgTTcCCGCCcCCAGAcAT-3′; Gene Tools; lowercase letters indicate mispairing nucleotides) or mRNA translation blocking CX3CL1-specific morpholino oligonucleotides (5′-TCCCCTTGCCGCCGCCAGAGAT-3′; Gene Tools) in EGM2MV media containing 6 µM Endo-Porter (Gene Tools). LECs were then incubated in exosome-free EGM2MV media with 7 ng/ml TNFα (R&D Systems) for 24 h. Basolateral LEC culture supernatant was collected, and EEV fractions were isolated as described above.

#### Calculations for morphometric and migration analyses

Fluorescence diameter ratio:

$$\frac{\sigma CD9}{\sigma podoplanin}$$

σ is the distance between fluorescence minima djacent to the vessel minus associated fluorescent maxima.

Chemotactic displacement:

$$\frac{Displacement\;upgradient}{Track\;length}$$

Speed:

$$\frac{Track\;length}{Time}$$

Migratory angle change:

$$\sqrt{{\left(\theta \left|t_{i}\right|-\theta \left|t_{i+1}\right|\right)}^{2}}$$

θ is the migratory angle at the given time point.

Chemotactic index = *cosΘ*

θ is the angle between a line directly upgradient and one to the endpoint of each cell’s track.

Circularity:

$$4\pi \cdot \left(\frac{Area}{Perimeter^{2}}\right).$$

### Microfluidic adhesion assay of human prostate carcinoma cells

For the adhesion assay, EEV fractions were isolated from 2.6 ml basolateral LEC culture supernatant and resuspended in 5 µl PBS per sample. An equally sized volume of EEV-free basolateral LEC culture supernatant was used as a control. Microfluidic capillaries (Cellix) were coated with EEV fractions or EEV-free supernatants at 4°C for 12 h, blocked with 10 mg/ml BSA in PBS for 1 h at 37°C, and washed with DMEM. PC3-ML human prostate carcinoma cells were grown in DMEM (10% FBS) to 80% confluency and were then trypsinized, washed, and resuspended at a concentration of 2.25 ×10^6^ cells/ml DMEM (10% FBS). 50 µl cell suspension was loaded into the reservoir wells of the microfluidic capillaries. Using the Mirus Evo Nanopump (Cellix), cells were exposed to a shear stress of 0.5 dynes/cm^2^, and time-lapse image series were acquired every 1 s with an exposure time of 0.344 s. Brightfield images were acquired with inverted DM IL light-emitting diode microscopes with 10× 0.22 NA HI Plan objectives and the SV-Timelapse software. Adherent cell tracks were quantitated semiautomatically with the tracking tool of Imaris imaging software. Cells that adhered for at least three consecutive seconds were considered adherent cell tracks.

### Transwell transmigration of human prostate carcinoma cells

EEV fractions from 3.9 ml basolateral LEC culture supernatant were resuspended in 10 µl DMEM per sample. An equally sized volume of EEV-free basolateral LEC culture supernatant was used as a control. PC3-ML human prostate carcinoma cells were grown in DMEM (10% FBS) to 80% confluency and were then trypsinized, washed, and resuspended at a concentration of 0.5 × 10^6^ cells/ml DMEM (10% FBS). EEV fractions or EEV-free supernatants were applied to the lower chambers of 12-well plates before the transfer of 2.5 × 10^5^ cancer cells to the upper 12-mm transwell cell culture inserts (12-µm pore size, 10^5^ pores/cm^2^; Corning). To investigate the involvement of CX3CL1 in EEV fraction-promoted cancer cell transmigration, mouse anti–human CX3CL1 neutralizing IgGs (R&D Systems) or mouse isotype control IgGs (R&D Systems) were applied simultaneously with the EEV fractions or the EEV-free supernatants to the lower chambers of the 12-well plates. Cancer cells were allowed to migrate through the pores of the 10-µm-thick polyethylene membrane into the lower chamber wells at 37°C and 5% CO_2_ for 24 h. Brightfield images of the entire lower chambers were acquired with inverted DM IL light-emitting diode microscopes with 4× 0.10 NA HI Plan objectives and the SV-Timelapse software. Transmigrated cells in the lower chambers were counted manually.

### Statistics

Prism 5 (GraphPad Software) was used to test the normality of the data and for subsequent parametrical two-tailed *t* tests (with Welch’s correction where necessary).

### Online supplemental material

Figure S1 shows immunohistological evidence for exosome accumulation in the perilymphatic stroma of inflamed tissues. Figure S2 shows the leakiness of primary LEC monolayers for 70-kD dextran and raw Western blot images of EEV fractions. Figure S3 shows heat maps of prominent protein signatures enriched in EEV fractions. Figure S4 shows that EEVs are taken up by MMDCs and that EEVs promote the directional migration of MMDCs in transwell migration assays and collagen migration assays. Figure S5 shows that CX3CR1 is expressed in cells recruited into lymphatic vessels of inflamed kidneys and in MMDCs and that targeted knockdown of CX3CL1 expressed in EEVs inhibits the MMDC-migration–promoting effects of EEVs in the transwell migration assay. Table S1 lists the significantly identified proteins from MS measurements of EEV fractions. Videos 1, 2, and 3 show endothelial monolayer transmigration assays with MMDCs in the presence of EEV-free supernatant, ss-EEV fractions, or TNFα−EEV fractions. Videos 4 and 5 show the collagen migration assay with MMDCs in the presence or absence of hCCL19 gradients. Videos 6, 7, and 8 show confined microenvironment migration assays with MMDCs in the presence of EEV-free supernatant, ss-EEV fractions, or TNFα-EEV fractions.

## Supplementary Material

Supplemental Materials (PDF)

Table S1 (Excel)

Video 1

Video 2

Video 3

Video 4

Video 5

Video 6

Video 7

Video 8
