# Supplementary material for: Lymphatic exosomes promote dendritic cell migration along guidance cues
Source: J Cell Biol. 2018 Jun 4;217(6):2205–21. doi: 10.1083/jcb.201612051 (PMC5987709; doi:10.1083/jcb.201612051)
Supplement: Supplemental Materials (PDF) [file JCB_201612051_sm.pdf]

Supplemental material

Brown et al., https://doi.org/10.1083/jcb.201612051

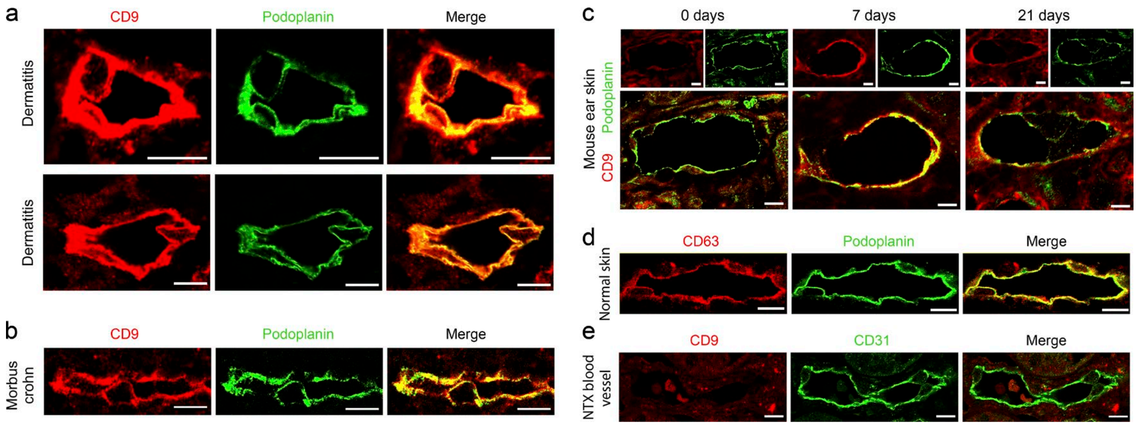

Figure S1. **Immunohistological evidence for exosome accumulation in the perilymphatic stroma of inflamed tissues (related to Fig. 1). (a–e)** Immunofluorescence of the lymphatic vessel marker podoplanin (a–d, green) or the panvascular marker CD31 (e, green) and the exosome markers CD9 (a–c and e, red) or CD63 (d, red). **(a)** Human dermal lymphatics of patients with allergic dermatitis (*n* = 5). **(b)** Human intestinal lymphatics of patients with Crohn's disease (*n* = 4). **(c)** Mouse dermal lymphatics (ear) with/without oxazolone induced hypersensitivity dermatitis. Days of treatment are indicated (*n* = 3). **(d)** Dermal lymphatics of normal human skin (*n* = 6). **(e)** Human renal blood vessel in renal transplant rejection (*n* = 14). NTX, renal kidney transplant. Bars, 10 µm.

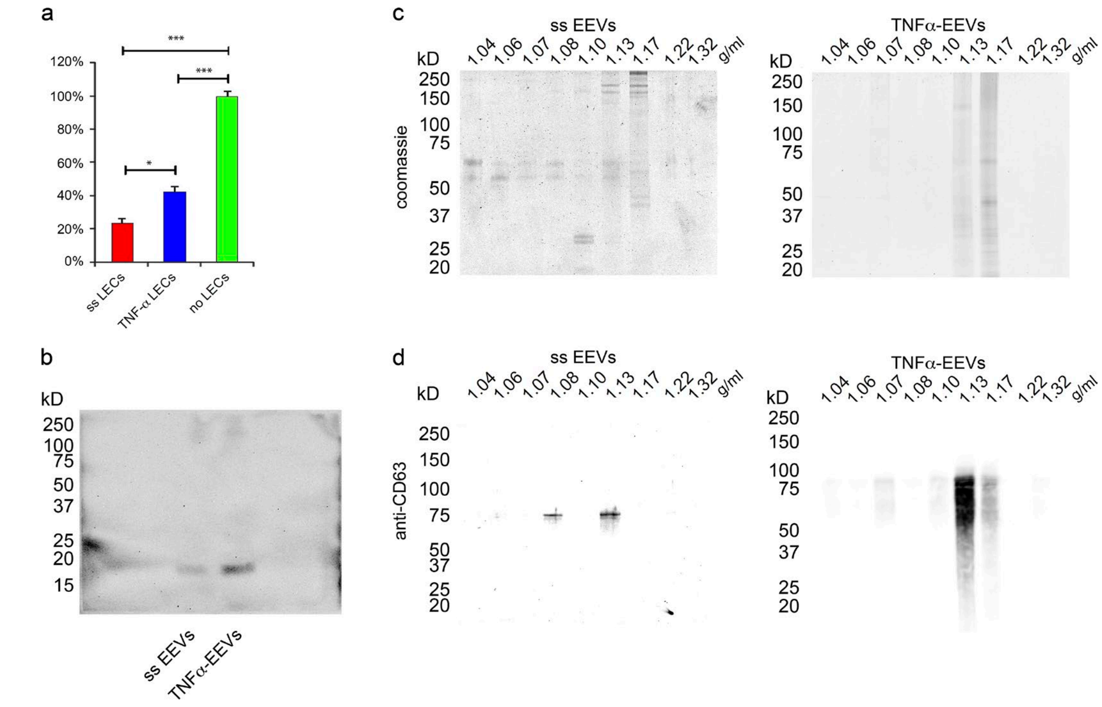

Figure S2. **Primary human LECs release EEVs (related to Fig. 2). (a)** Relative leakage as determined by mean fluorescence intensity (MFI) of 70-kD FITC-dextran from the upper cell culture transwell insert through confluent monolayers of LECs into the lower chamber wells was determined in the absence or presence of 7 ng/ml TNFα with a fluorescent plate reader. 100% leakage was determined in the absence of LEC monolayers (*n* = 5; unpaired two-tailed *t* test). Values represent means ± SEM. **(b)** Original (Fig. 2 f) anti-CD9 immunoblot of EEV fractions from equal volumes of basolateral culture supernatants of ss and TNFα-stimulated LECs. **(c)** Original (Fig. 2 h) Coomassie blue–stained electrophoresis gel of density gradient centrifugation fractions of EEV fractions isolated from basolateral culture supernatants of ss (left) and TNFα-stimulated (right) LECs. **(d)** Original (Fig. 2 i) anti-CD63–stained immunoblot of density gradient centrifugation fractions of EEV fractions isolated from basolateral culture supernatants of ss (left) and TNFα-stimulated (right) LECs. *, P ≤ 0.05; ***, P ≤ 0.001.

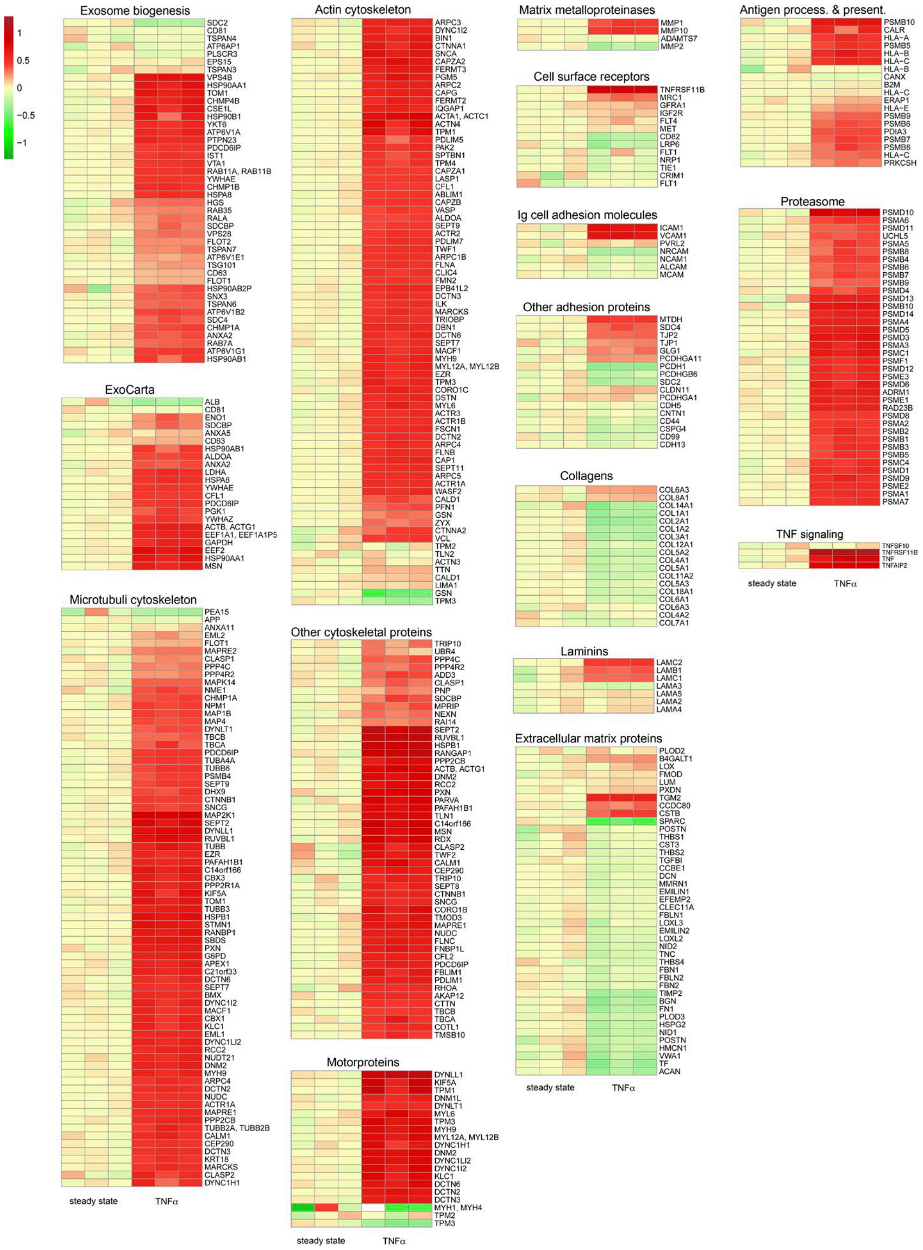

Figure S3. **Proteomic profiling reveals migration-promoting protein signature in TNFα EEV fractions (related to Fig. 3).** EEV fractions from basolateral cell culture supernatants of ss or TNFα-stimulated LECs (n = 3) were proteomically profiled with TMT-based LC-MSMS analysis. Heat maps of significantly ($P < 0.05$) quantified proteins were compiled into the following biologically relevant protein clusters: Exosome biogenesis, ExoCarta marker proteins, microtubular cytoskeleton, actin cytoskeleton, other cytoskeletal proteins, motor proteins, matrix metalloproteinases, cell surface receptors, Ig superfamily cell adhesion molecules, other adhesion proteins, collagens, laminins, ECM proteins, antigen processing and presentation proteins, proteasomal proteins, and TNF signaling. Three left lanes: Replicate EEV fractions from ss LECs. Right three lanes: Replicate EEV fractions from TNFα-stimulated LECs. Values in heat maps are $\log_{10}$ of interprotein abundance of a given sample divided by the mean abundance of the three ss samples.

https://doi.org/10.1083/jcb.201612051

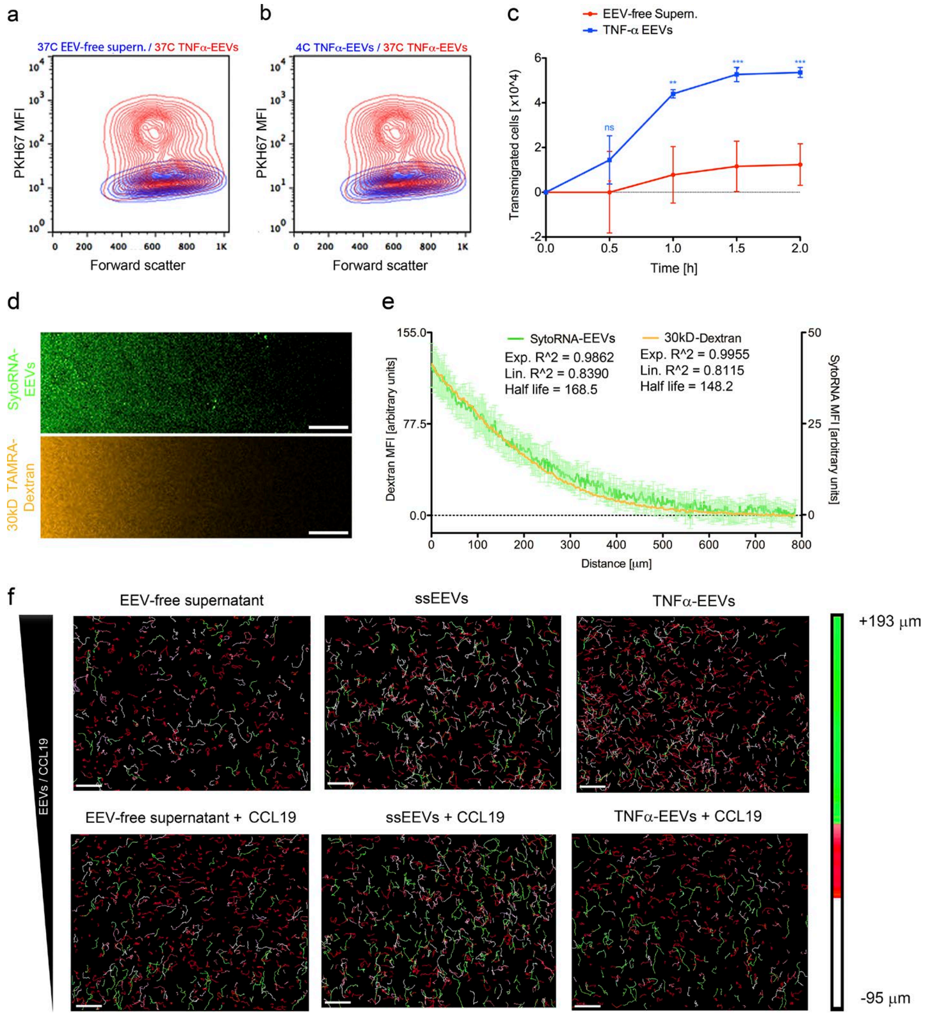

Figure S4. **EEV fractions promote the directional migration of MMDCs in response to guidance cues in vitro and ex vivo by inducing formation of cellular protrusions (related to Fig. 4). (a)** Flow cytometry contour plots of MMDCs incubated for 60 min with PKH67-stained EEV-free supernatants (blue) or PKH67-stained TNFα-EEV fractions (red) at 37°C (*n* = 3; unpaired two-tailed *t* test). X axis indicates forward scatter. Y axis indicates mean fluorescence intensity (MFI) of PKH67 dye. **(b)** Flow cytometry contour plots of MMDCs incubated for 60 min with PKH67-stained TNFα-EEV fractions at 4°C (blue) or PKH67-stained TNFα-EEV fractions at 37°C (red; *n* = 3; unpaired two-tailed *t* test). X axis indicates forward scatter. Y axis indicates mean fluorescence intensity of PKH67 dye. **(c)** Quantitation of transmigrated MMDCs from the upper cell culture insert into the lower chamber well of a transwell assay. MMDCs and EEV-free supernatants (red) or TNFα-EEV fractions (blue) were loaded simultaneously into the upper cell culture inserts (*n* = 3; unpaired two-tailed *t* test). **(d)** Fluorescence images of exponential gradients of SYTO RNASelect–labeled EEV fractions (top) or tetramethylrhodamine (TAMRA)-labeled 30-kD dextrans (bottom) in 3D collagen matrices (*n* = 3). **(e)** Fluorescence profiles of exponential gradients of SYTO RNASelect–labeled EEV fractions (green) or TAMRA-labeled 30-kD dextrans (yellow) in 3D collagen matrices (*n* = 3). X axis indicates cross-sectional distance. Y axis indicates SYTO RNASelect or TAMRA-specific fluorescence intensities. **(f)** Migratory cell tracks of automatically segmented MMDCs in 3D collagen matrix migration assays. Cells were exposed to gradients of EEV-free supernatants, ss-EEV fractions, TNFα-EEV fractions, EEV-free supernatants plus CCL19, ss-EEV fractions plus CCL19, or TNFα-EEV fractions plus CCL19 (*n* = 3). Color coding: green, positive chemotactic displacement; red, neutral chemotactic displacement; white, negative chemotactic displacement. Bars, (d) 100 µm; (f) 200 µm. Values represent means ± SEM. ns, P > 0.05; **, P ≤ 0.01; ***, P ≤ 0.001.

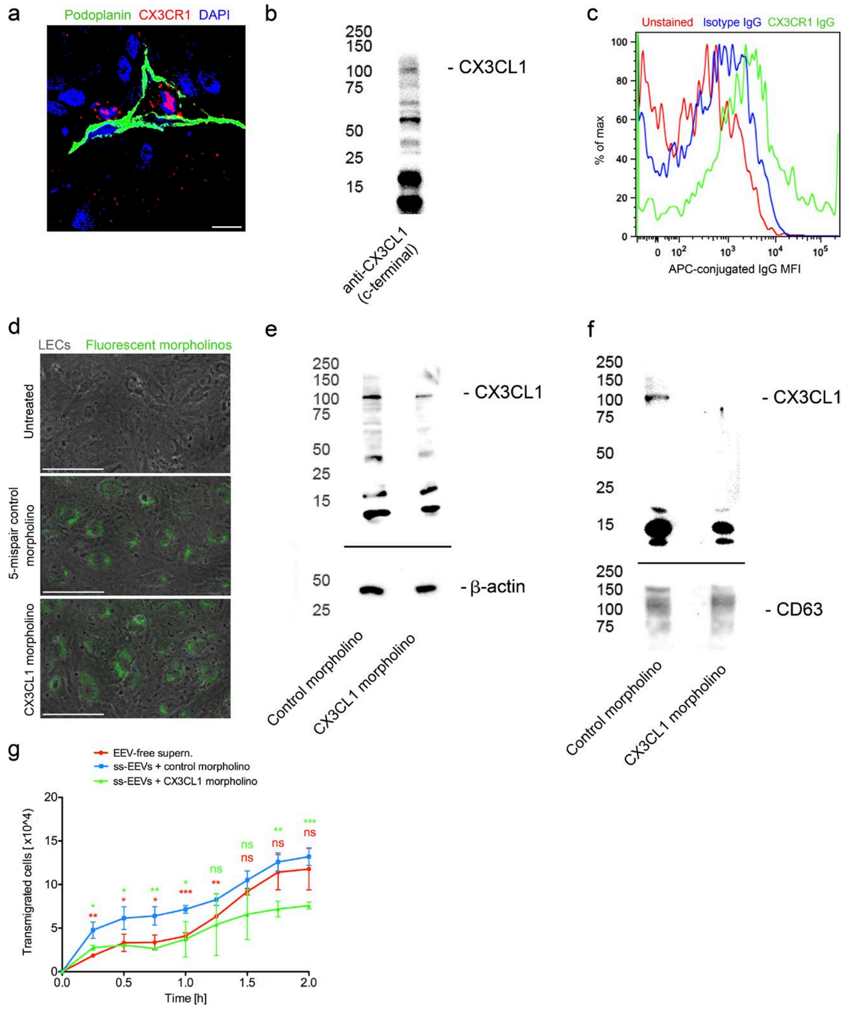

Figure S5. **Induction of protrusion formation and enhancement of directional migration by TNFα-EEV fractions is dependent on GPCR signaling and CX3CL1 (related to Fig. 6)**. **(a)** Immunofluorescence of the lymphatic vessel marker podoplanin (green) and CX3CR1 (red) in human renal transplant rejections. Cell nuclei are stained with DAPI (*n* = 6). **(b)** Anti-CX3CL1 immunoblot of whole-cell lysates of TNFα-stimulated LECs probed with an antibody to the C terminus of CX3CL1 (*n* = 3). **(c)** Flow cytometry histogram of unstained (red), APC-conjugated isotype IgG-stained (blue), and APC-conjugated anti-CX3CR1 IgG-stained (green) human mature MMDCs. X axis indicates percentage of maximum. Y axis indicates mean fluorescence intensity (MFI) of APC-conjugated IgG (*n* = 4). **(d)** Composite transmitted light and epifluorescence images of LEC monolayers (gray) that were untreated (top), treated with fluorescein-labeled 5-mispair control morpholinos (green; middle), or treated with fluorescein-labeled CX3CL1 morpholinos (green; bottom; *n* = 3). Bars: (a) 10 µm; (d) 100 µm. **(e)** Anti-CX3CL1 (C-terminal; top) and anti–β-actin (loading control; bottom) immunoblots of whole-cell lysates of TNFα-stimulated LECs treated with 5-mispair control morpholinos or CX3CL1 morpholinos (*n* = 3). **(f)** Anti-CX3CL1 (C-terminal; top) and anti-CD63 (loading control; bottom) immunoblots of TNFα-EEV fractions derived from LECs treated with 5-mispair control morpholinos or CX3CL1 morpholinos (*n* = 3). Molecular masses are given in kilodaltons. **(g)** Quantitation of transmigrated MMDCs from the upper cell culture insert into the lower chamber well of a transwell assay. MMDCs were loaded together with EEV-free supernatants or ss-EEV fractions derived from 5-mispair control morpholino oligonucleotide–treated LECs or CX3CL1-specific morpholino oligonucleotide–treated LECs into the upper cell culture insert (*n* = 2; unpaired two-tailed *t* test). Values represent means ± SEM. ns, P > 0.05; *, P ≤ 0.05; **, P ≤ 0.01; ***, P ≤ 0.001.

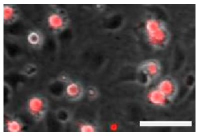

Video 1. **Endothelial monolayer transmigration assay with fluorescently stained human mature MMDCs migrating in the presence of EEV-free supernatant from TNFα-stimulated LECs on primary LECs that were transduced with a lentiviral construct encoding CCL21 (related to Fig. 4).** Brightfield and fluorescence images were acquired every 2.2 s for 40 min, and the last 30 min were used for generating the image sequence (frame rate, 90 frames/s; playback speed, 3.3 min/s). Transmigrated MMDCs have a spread out morphology and show decreased red fluorescence intensity. Bar, 50 µm.

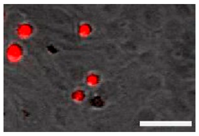

Video 2. **Endothelial monolayer transmigration assay with fluorescently stained human mature MMDCs migrating in the presence of EEV fractions from ss LECs on primary LECs that were transduced with a lentiviral construct encoding CCL21 (related to Fig. 4).** Brightfield and fluorescence images were acquired every 2.2 s for 40 min, and the last 30 min were used for generating the image sequence (frame rate, 90 frames/s; playback speed, 3.3 min/s). Transmigrated MMDCs have a spread out morphology and show decreased red fluorescence intensity. Bar, 50 µm.

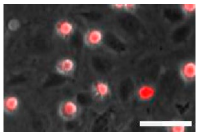

Video 3. **Endothelial monolayer transmigration assay with fluorescently stained human mature MMDCs migrating in the presence of EEV fractions from TNFα-stimulated LECs on primary LECs that were transduced with a lentiviral construct encoding CCL21 (related to Fig. 4).** Brightfield and fluorescence images were acquired every 2.2 s for 40 min, and the last 30 min were used for generating the image sequence (frame rate, 90 frames/s; playback speed, 3.3 min/s). Transmigrated MMDCs have a spread out morphology and show decreased red fluorescence intensity. Bar, 50 µm.

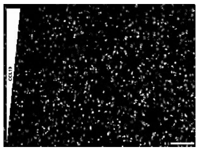

Video 4. **3D collagen matrix migration assay with human mature MMDCs migrating along a CCL19 gradient (related to Fig. 5).** Brightfield images of MMDCs were acquired every 3 min for 153 min (frame rate, 5.7 frames/s; playback speed, 17 min/s). For better visualization, the gray values were inverted, and the background was subtracted by using Fiji imaging software. The CCL19 concentration is highest on the upper edge, and exponentially decreases toward the lower edge. Bar, 100 µm.

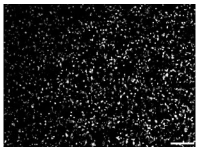

Video 5. **3D collagen matrix migration assay with human mature MMDCs migrating in the absence of a chemotactic gradient (related to Fig. 5).** Brightfield images of MMDCs were acquired every 3 min for 153 min (frame rate, 5.7 frames/s; playback speed, 17 min/s). For better visualization, the gray values were inverted, and the background was subtracted by using Fiji imaging software. Bar, 100 µm.

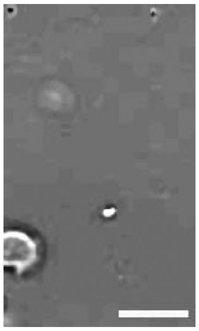

Video 6. **Confined microenvironment migration assay with human mature MMDCs migrating in the presence of EEV-free supernatant of TNFα-stimulated LECs (related to Fig. 5).** Brightfield images of MMDCs were acquired every 1 min for 106 min, and the last 77 min were used for generating the image sequence (frame rate, 7 frames/s; playback speed, 7 min/s). For better visualization, the gray values were inverted by using Fiji imaging software. Bar, 15 µm.

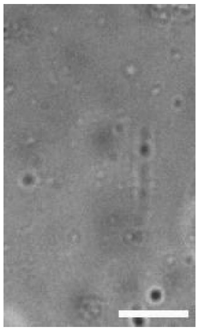

Video 7. **Confined microenvironment migration assay with human mature MMDCs migrating in the presence of EEV fractions of ss LECs (related to Fig. 5).** Brightfield images of MMDCs were acquired every 1 min for 106 min, and the last 77 min were used for generating the image sequence (frame rate, 7 frames/s; playback speed:, 7 min/s). For better visualization, the gray values were inverted by using Fiji imaging software. Bar, 15 µm.

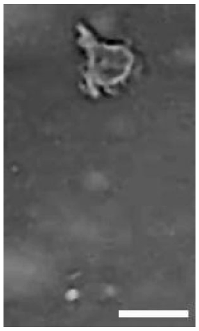

Video 8. **Confined microenvironment migration assay with human mature MMDCs migrating in the presence of EEV fractions of TNFα-stimulated LECs (related to Fig. 5).** Brightfield images of MMDCs were acquired every 1 min for 106 min, and the last 77 min were used for generating the image sequence (frame rate, 7 frames/s; playback speed:, 7 min/s). For better visualization, the gray values were inverted by using Fiji imaging software. Bar, 15 µm.

Table S1 is a separate Excel document showing significantly identified proteins from MS measurements of EEV fractions.
